# Insights into the Role of Transcriptional Gene Silencing in Response to Herbicide-Treatments in *Arabidopsis thaliana*

**DOI:** 10.3390/ijms22073314

**Published:** 2021-03-24

**Authors:** Catarine Markus, Ales Pecinka, Aldo Merotto

**Affiliations:** 1 Department of Crop Science, Federal University of Rio Grande do Sul, Porto Alegre, RS 91540-000, Brazil; aldo.merotto@ufrgs.br; 2 Department of Plant Breeding and Genetics, Max Planck Institute for Plant Breeding Research, D-50829 Cologne, Germany; 3 Institute of Experimental Botany, Czech Academy Science, Centre of the Region Haná for Biotechnological and Agricultural Research, Šlechtitelů 31, CZ-77900 Olomouc, Czech Republic

**Keywords:** epigenetics, herbicide resistance, chromatin mutants, ROS1, imazethapyr, glyphosate, 2,4-D

## Abstract

Herbicide resistance is broadly recognized as the adaptive evolution of weed populations to the intense selection pressure imposed by the herbicide applications. Here, we tested whether transcriptional gene silencing (TGS) and RNA-directed DNA Methylation (RdDM) pathways modulate resistance to commonly applied herbicides. Using *Arabidopsis thaliana* wild-type plants exposed to sublethal doses of glyphosate, imazethapyr, and 2,4-D, we found a partial loss of TGS and increased susceptibility to herbicides in six out of 11 tested TGS/RdDM mutants. Mutation in *REPRESSOR OF SILENCING 1* (*ROS1*), that plays an important role in DNA demethylation, leading to strongly increased susceptibility to all applied herbicides, and imazethapyr in particular. Transcriptomic analysis of the imazethapyr-treated wild type and *ros1* plants revealed a relation of the herbicide upregulated genes to chemical stimulus, secondary metabolism, stress condition, flavonoid biosynthesis, and epigenetic processes. Hypersensitivity to imazethapyr of the flavonoid biosynthesis component *TRANSPARENT TESTA 4* (*TT4*) mutant plants strongly suggests that ROS1-dependent accumulation of flavonoids is an important mechanism for herbicide stress response in *A. thaliana*. In summary, our study shows that herbicide treatment affects transcriptional gene silencing pathways and that misregulation of these pathways makes Arabidopsis plants more sensitive to herbicide treatment.

## 1. Introduction

Weeds are among the main factors reducing crop production worldwide [1]. Herbicide application currently represents the most cost-effective method of weed control; however, the application of herbicides to the large natural weed populations may result in the selection of resistant individuals and subsequently whole populations [2]. Herbicide resistance is an important challenge in modern agriculture since new cases are being described every year [3]. In some regions, this problem has reached widespread levels and is a remarkable example of rapid, human-influenced adaptation with a large impact on crop production costs and decreasing food production [4,5,6]. Herbicide resistance in weeds is defined as the “survival of a segment of the population of a plant species following an herbicide dose lethal to the normal population” [7]. It is important to note that in Weed Science, the resistance is contrasted with tolerance, which is characterized by “survival of the normal population of a plant species following an herbicide dosage lethal to other species” [7].

The two major mechanisms of resistance to herbicides are target-site resistance (TSR) and non-target-site resistance (NTSR) [8]. TSR is caused primarily by DNA mutations that result in amino acid changes and subsequently structural changes to herbicide-binding sites, or increased expression of target proteins [9,10,11]. The less well-understood NTSR include genes affecting physiological and/or developmental traits such as decreased herbicide absorption, translocation and/or enhanced detoxification [12,13,14,15]. Enhanced detoxification is the most studied for NTSR and is caused by enzymatic systems detoxifying xenobiotics in plants, mediated mainly by cytochrome P450 monooxygenases (P450s), glutathione transferases (GSTs) and/or herbicide exclusion attributable to ATP-dependent (ATP-binding cassette (ABC)) transporters [13]. The NTSR tends to be more complex due to its quantitative nature that is controlled by the accumulation of the minor effect resistance alleles. The mechanisms of NTSRs are not fully understood, and their components are often part of plant stress responses, which evolve over time [16].

It is hypothesized that also epigenetic changes may be responsible for the rapid occurrence of herbicide resistance [17,18,19]. Epigenetic regulation has been linked with many developmental processes, stress responses, and genome functions [20,21]. It consists of chemical modifications of DNA and/or histone proteins in chromatin, which result in different expression patterns of otherwise identical DNA sequences [22]. Two major epigenetic repressive pathways have been described in plants. The Polycomb Repressive Complexes (PRC) install histone H3 lysine 27 tri-methylation via PRC2 and histone H2B lysine 118/119 ubiquitination via PRC1 [23]. The PRC-based silencing regulates developmentally controlled genes. The second module is specialized in constitutive repression of transposable elements and other repetitive DNAs by installing a high density of DNA methylation in all sequence contexts [24]. The silencing machinery is navigated to its target sites by small RNAs with perfect sequence homology and therefore it is known as RNA-directed DNA methylation (RdDM). Once the silencing is established, it is fixed by a series of chromatin biochemical and structural modifications leading to stable transcriptional gene silencing (TGS) [25].

A study with atrazine in rice (*Oryza sativa*) indicated that epigenetic alterations were associated with the activation of specific genes responsible for atrazine detoxification [26]. In bread wheat (*Triticum aestivum*), applications of increasing glyphosate concentrations raised the levels of global DNA methylation from 28% to 74% [27]. Additionally, sublethal glyphosate doses induced dose-dependent differentially methylated regions across the Arabidopsis genome [18]. Even though some studies address the epigenetic relationship with herbicide resistance [18,26,27,28,29], the understanding of how it occurs is only at its beginning. We hypothesized that epigenetic regulation via DNA methylation may modulate herbicide stress response by influencing the expression of specific genes as implicated in weed adaptation to other stresses. To this end, we analyzed the effects of three commonly used herbicides glyphosate, imazethapyr and 2,4-D, with different mechanisms of action, to verify their impact on RdDM and TGS regulation. Glyphosate is an inhibitor of 5-ENOLPYRUVYLSHIKIMATE-3-PHOSPHATE SYNTHASE (EPSPS; AT1G48860); imazethapyr belongs to inhibitors of ACETOLACTATE SYNTHASE (ALS; AT3G48560), and finally, 2,4-D (2,4-dichlorophenoxyacetic acid) is a synthetic auxin, which mimics naturally the plant hormone indole-3-acetic acid (IAA), the principal natural auxin in higher plants [30]. The present study is based on *Arabidopsis thaliana*, which has a wide range of well-characterized mutants and epigenetic regulators [31]. This study aimed to identify whether epigenetic mechanisms, such as TGS/RdDM, can be involved in the response of *A. thaliana* to herbicide stress, and whether these changes may prove to afford critical insight into the known mechanisms of herbicide resistance. Here we show that DNA demethylation or hypermethylation negatively influence the response of *A. thaliana* plants to the herbicides glyphosate, 2,4-D, and especially to imazethapyr. This sheds new light on a potential additional adaptive mechanism for herbicide resistance.

## 2. Results

### 2.1. Sublethal Doses of Glyphosate, Imazethapyr and 2,4-D Weaken TGS

Initially, we established experimental conditions for sublethal treatments of Arabidopsis wild-type plants with various herbicides. Data of plant injury and dry weight were fitted to the sigmoidal logistic regression model with three parameters, and allowed identifying the appropriate sublethal doses for each herbicide treatment for Col-0 wild-type plants (Table S1, Figures S1 and S2). The sublethal dose was selected based on the biomass reduction between 65–85% but allowing for plant survival and reproduction (Figures S1 and S2). Based on that, the selected doses for glyphosate, imazethapyr, and 2,4-D were 72, 10.6, and 40.3 g ha^−1^, corresponding to approximately 10%, 10%, and 5% field rate of each herbicide, respectively.

**Figure 1:**
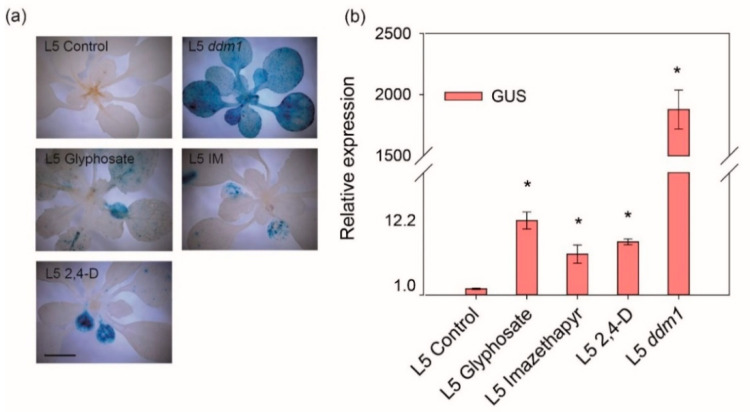
Herbicide stress effect on epigenetically regulated transcription. (**a**) Representative *β-Glucuronidase* (*GUS*)-stained shoots of the plants of line L5, 48 h after treatment with glyphosate, imazethapyr (IM) and 2,4-D, non-treated plant (L5 control) and L5 *ddm1* plant serving as a positive control. Plants line L5 are carrying a single insert of a multicopy *35S Cauliflower Mosaic Virus Promoter* driven *GUS* (*Pro35S::GUS*) locus in L5/6b5 line, gene suppressed by transcriptional gene silencing (TGS), all mutants are in the Col-0 background. Scale bar = 1 cm. (**b**) Relative transcript abundance of *GUS* in L5 measured by RT-qPCR 48 h after the treatment. Error bars indicate the standard deviation of the three biological replicates. Statistically significant differences relative to L5 control are indicated by asterisks (*t*-test, *p* ≤ 0.05); * compared to L5 Control.

To investigate whether the herbicide treatments influence transcriptional gene silencing, we used multicopy *35S Cauliflower Mosaic Virus Promoter* driven *β-Glucuronidase* (*Pro35S::GUS*) reporter gene in L5/6b5 line [32,33,34]. The 21 days old L5 plants were exposed to sublethal doses of glyphosate, imazethapyr and 2,4-D and screened for activation of the reporter gene (Figure 1a). According to the histochemical staining, the applied doses of herbicides induced GUS activation and accumulation in the cotyledons and to a smaller extent also in the true leaves (Figure 1a). The amount of signal in true leaves was variable for different herbicides. These results were confirmed by RT-qPCR, which revealed 12.2, 6.7, and 8.7-fold higher amount of *GUS* transcript in glyphosate, imazethapyr, and 2,4-D treated plants relative to mock-treated control, respectively (*t*-test, *p* ≤ 0.05) (Figure 1b). However, the herbicide-induced *GUS* activation was weak after herbicide treatment when compared to the strong effect of epigenetic mutant for *DECREASED IN DNA METHYLATION 1* chromatin remodeling factor (L5 *ddm1*) plants, which revealed >2000-fold increase *GUS* activation (Figure 1b). Hence, the differences between individual herbicide treatments are negligible. These results indicated that the herbicide treatments at least partially interfere with TGS.

### 2.2. The Response of Chromatin Mutants to the Herbicide Treatments

**Figure 2:**
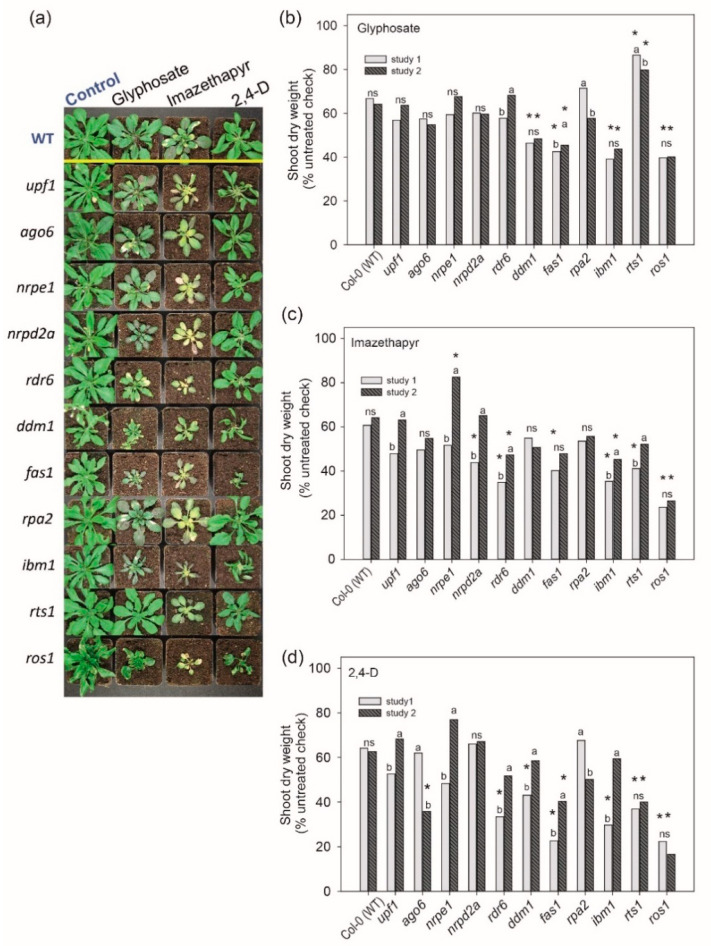
(**a**) Effect of sublethal doses of glyphosate, imazethapyr and 2,4-D on *Arabidopsis thaliana* wild type (WT) and selected chromatin mutants 10 days after treatment (DAT). (**b**–**d**) Shoot dry weight from treated plants compared to non-treated plants (% untreated check) after (**b**) glyphosate, (**c**) imazethapyr and (**d**) 2,4-D treatment. The mean of each study (1 and 2) was composed by three biological replicates, studies 1 and 2 followed by different letter differ significantly by Tukey’s test (*p* ≤ 0.05); ns = nonsignificant. Treated mutants followed by asterisk differ significantly from treated WT, according to Tukey’s test (*p* ≤ 0.05); * compared to WT.

To test whether the TGS contributes to gene regulation under herbicide stress, we selected mutants in 1 TGS/RdDM representative genes, exposed them to the above-defined sublethal doses of herbicides and analyzed their growth (Figure 2). Measurements of shoot dry weight and leaf length 10 days after treatment (DAT) showed that some mutants responded differently compared to wild type (*p* ≤ 0.05) (Figure 2 and Figure S3). The mutant plants in *RNA-DEPENDENT RNA POLYMERASE* (*rdr6*), *FASCIATA 1* (*fas1*)*, HISTONE DEACETYLASE 6* as known as *RNA-MEDIATED TRANSCRIPTIONAL SILENCING* (*rts1*), *INCREASE IN BONSAI METHYLATION 1* (*ibm1*), and *ddm1* differed from the wild-type plants for at least one of the tested herbicides in two independent experiments, each with three biological replicates (Figure 2 and Figure S3). The *rdr6* plants had increased susceptibility to imazethapyr, *ddm1* showed reduced shoot dry weight in response to glyphosate treatment, *fas1* plants were more susceptible to glyphosate and 2,4-D, and *ibm1* had increased susceptibility to glyphosate and imazethapyr in both studies and for 2,4-D in the first study (Figure 2). Surprisingly, hypomorphic mutant plants of *REPRESSOR OF SILENCING 1* (*ROS1*) showed high susceptibility to all herbicides, represented by ≈35%, 55%, and 62% reduction in shoot dry weight (Figure 2).

**Figure 3:**
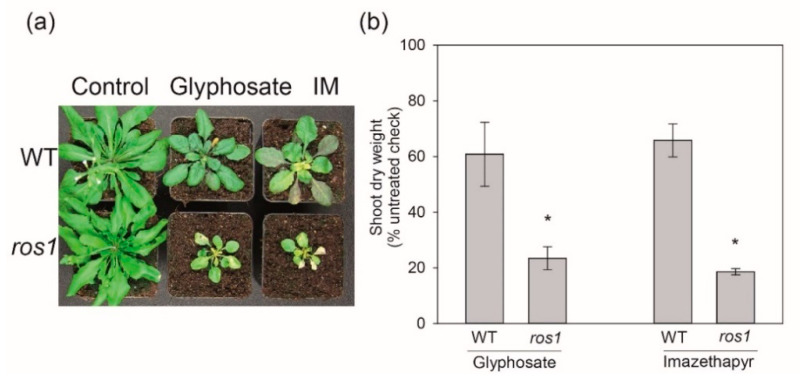
(**a**) Visual effect (**b**) shoot dry weight of *A. thaliana* Col-0 wild type (WT) and *ros1* mutant corresponding to study 3, 10 days after treatment (DAT) of glyphosate and imazethapyr (IM). Error bars indicate the standard deviation of four biological replicates, statistically significant differences relative to WT is indicated by asterisk (*t*-test, *p* ≤ 0.05); * compared to WT.

**Figure 4:**
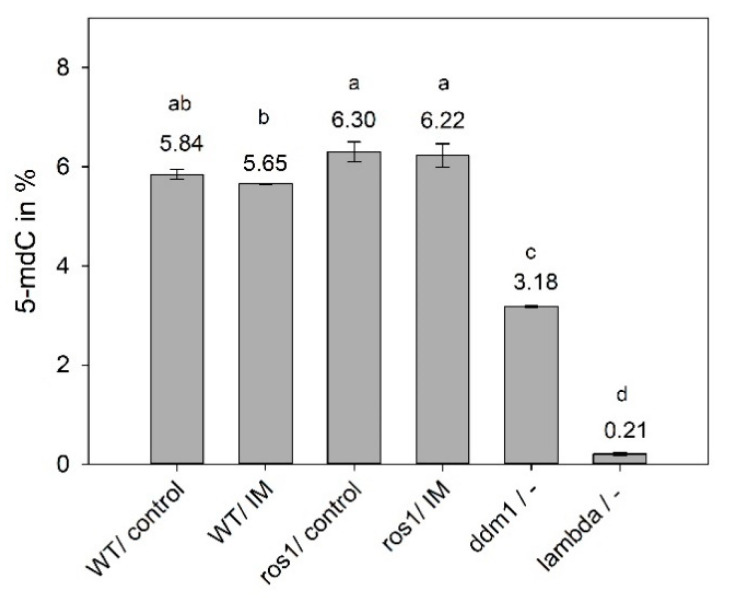
HPLC measurement of global 5-mdC (%) in wild type (WT) and *ros1*, in the control condition and after treatment with imazethapyr (IM), 48 h after treatment. *ddm1* and lambda phage DNA were used as low and high DNA methylation controls, respectively. Error bars denote standard deviations from three biological replicates. Means followed by a different letter differ significantly according to Tukey’s test (*p* ≤ 0.05).

To validate the results for *ros1*, the experiment was repeated independently three more times with glyphosate and imazethapyr (Figure 3). The experiments present similar results, with the following exception for glyphosate in study 4, were *ros1* presented intermediary susceptibility to glyphosate (studies 4 and 5 not shown). This suggests that ROS1 is necessary for the normal plant tolerance to these herbicides. ROS1 is a plant-specific DNA glycosylase, which removes 5-methylcytosine (5mC) by a noncanonical base excision repair mechanism and thus counteracts the silencing activity of TGS/RdDM at specific loci [35,36]. To test whether ROS1 controlled methylation plays a role in herbicide resistance, we analyzed global genomic DNA methylation levels using HPLC (Figure 4). Control-treated wild-type plants contained 5.8% (±0.10) and *ros1* plants 6.3% (±0.20) of methylated cytosines (F test, *p* ≤ 0.05), confirming that loss of *ROS1* function leads to DNA hypermethylation [37]. In contrast, *ddm1* and lambda phage DNA, representing low methylation controls, had only 3.18 (±0.02%) and 0.21 (±0.03%) of 5mCs, respectively. After imazethapyr treatment, DNA methylation was 5.65% (±0.01) in wild type and significantly different to 6.22% (±0.24) in *ros1* plants (Figure 4). Although the differences may seem small, we should consider that they represent % from the whole genome (ca. 157 mio bps) and thus mean tens to hundreds of thousands of cytosines being affected. In more details, about 18% of the Arabidopsis genome are Cs [38]. Hence, 5.84% mCs corresponds to 1,650,384 mCs. After imazethapyr treatment *ros1* presented 161,082 mCs more than in WT/IM. This indicated ROS1 as an interesting candidate for further analysis. The mutant *ddm1* showed to affect more intensely the mCs, presenting 751,716 mCs less than in WT. It is important to mention that the responses in this study are congruent with line L5 study, where the herbicide showed less *GUS* activation compared to *ddm1* (Figure 1).

### 2.3. Massive Transcriptional Changes Upon Imazethapyr Treatment

To determine candidate genes involved in the increased susceptibility of *ros1* plants to imazethapyr, we monitored transcript levels of genes expressed at 48 h after control and imazethapyr treatments in wild type and *ros1* plants by RNA-sequencing. Each sample was sequenced as a biological duplicate and a total of 163,214,163 short reads were mapped to the TAIR10 reference genome (Table S2). Approximately 97% of wild type total reads aligned to the reference genome, while it was about 88% for *ros1* (Table S2). The initial analysis and visualization of the gene expression data produced by Cuffdiff were performed by using CummeRbund (Figure S4).

**Figure 5:**
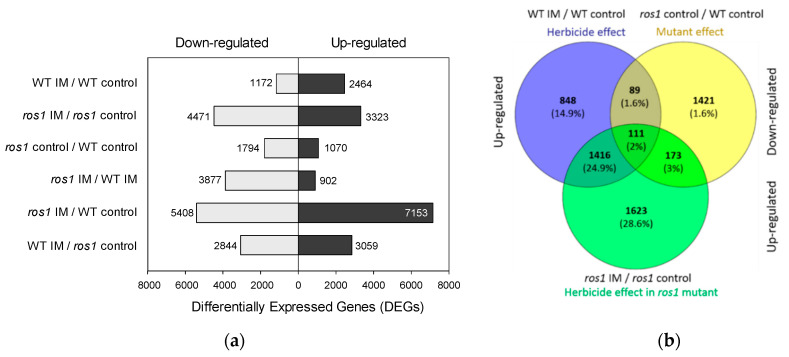
(**a**) Differentially expressed genes (DEGs) in response to imazethapyr (IM) and in the control condition in wild type (WT) and ros1 mutant. The number of up and downregulated genes is represented in black and gray bars, respectively. The differences in gene expression were obtained based on the Log2 Fold Change ≥2 and adjusted *p*-value < 0.05 in Cuffdiff. (**b**) Venn diagrams showing the overlap of genes comparing genes inducted by IM in WT (herbicide effect—blue circle), with repressed genes in ros1 (mutant effect—yellow circle) and effect of IM in ros1 (herbicide effect on mutant—green circle).

Results from the volcano plot demonstrated that a large number of genes were significantly (*p*-values < 0.05) differentially expressed between the pairs of conditions (Figure S4b). There were 2464 significantly upregulated and 1172 significantly downregulated differentially expressed genes (DEGs) in response to imazethapyr in wild-type plants (adjusted *p*-value < 0.05 in Cuffdiff; Figure 5a). In *ros1* plants, 4471 DEGs were downregulated and 3323 DEGs upregulated in response to imazethapyr (adjusted *p*-value < 0.05 in Cuffdiff; Figure 5a). Other possible comparisons showing up and downregulated genes are presented in Figure 5a. Out of the 2464 DEGs upregulated by imazethapyr treatment in wild type, 200 genes were controlled (presumably repressed) by *ROS1* as indicated by their downregulation in control *ros1* plants (Figure 5b). Out of 3323 DEGs upregulated by the combination of imazethapyr with *ros1* mutation, 26.9% (*n* = 1527) were in common with imazethapyr-induced genes in the wild type. Therefore, 89 genes appear to be of high interest based on their induction by imazethapyr in the wild type and downregulation in *ros1* mutant plants (Figure 5b).

### 2.4. Gene Ontology (GO) Analysis Identifies Enrichment of Secondary Metabolism and Flavonoid Biosynthesis Genes

To associate misregulated genes with specific biological processes, we performed GO enrichment analysis (Figure S5). Singular enrichment analysis (SEA) revealed an overrepresentation of genes from 32 biological processes in response to imazethapyr treatment in wild-type plants (Figure S6). The majority of overrepresented genes were linked to “response to stimulus” (including chemical stimulus), “stress” and “secondary metabolism” (Figure S6). The significant enrichment of included subcategories was more informative, for instance, “secondary metabolism”, where imazethapyr showed to affect genes potentially related to herbicide metabolism and detoxification, e.g., *GLUTATHIONE S-TRANSFERASE (CLASS PHI) 5* (*GSTF5*, AT1G02940), *GLUTATHIONE S-TRANSFERASE TAU 25* (*GSTU25*, AT1G17180) and *GLUTATHIONE S-TRANSFERASE PHI 12* (*GSTF12*, AT5G17220). Additionally, genes involved in the subcategory “flavonoid biosynthesis” were identified, such as *CHALCONE-FLAVANONE ISOMERASE FAMILY PROTEIN* (*CHIL*, AT5G05270), *FLAVANONE 3-HYDROXYLASE* (*F3H*; AT3G29590), *TRANSPARENT TESTA 4* (*TT4*, AT5G13930)*, TRANSPARENT TESTA 5* (*TT5*, AT3G55120), and *TRANSPARENT TESTA 7* (*TT7*, AT5G07990).

Downregulated genes also presented a similar division of classification in categories of cellular components, biological processes, and molecular function compared to upregulated genes (Figure S7). However, SEA revealed that subcategories of genes repressed after imazethapyr treatment were mainly involved in the regulation of “cell cycle”, “response to endogenous stimulus”, “response to gibberellin”, “anatomical structure”, and “morphogenesis” (Figure S8). Additionally, several genes encoding “components of chloroplast”, “light stimulus” and “photosystems” were downregulated, indicating reduction in photosynthesis compounds and changes in the expression of genes involved in central energy pathways due to the application of imazethapyr (Figure S8).

To understand the effects of imazethapyr on epigenetic regulation, putative genes involved in this process were analyzed comparing the wild-type control with the wild-type treated plants. Imazethapyr treatment caused significant (adjusted *p*-value < 0.05) upregulation of 40 genes and downregulation of 33 genes controlled by TGS according to modification data presented in Jacobsen lab epigenome browser (http://genomes.mcdb.ucla.edu/AthBSseq/, accessed on 8 January 2021) (Table S3).

#### (GO) Analysis of DEGs—Genes Putatively Involved with NTSR

**Figure 6:**
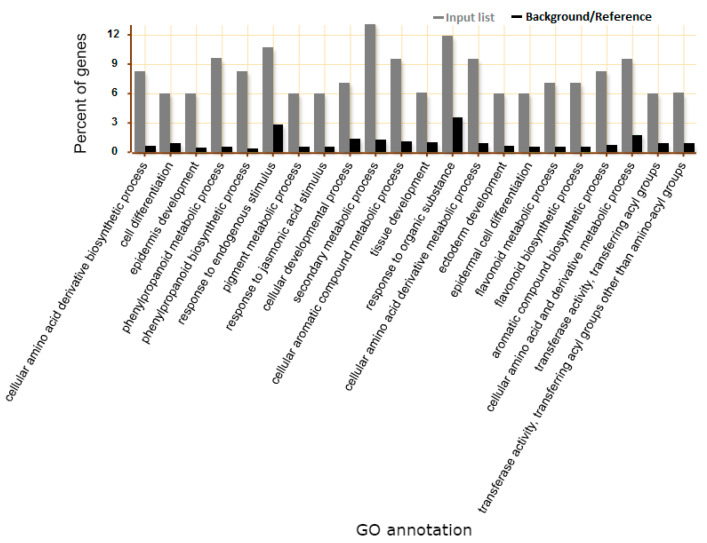
Detailed information of biological process representing the percent of genes involved in biological process pathways of 89 genes of interest induced by imazethapyr (IM) in wild type (WT) and downregulated in *ros1* mutant, performed by using singular enrichment analysis (SEA) in AgriGo. Gray and black bars indicate the percent of genes related to the input list and the percent of genes compared to genome reference, respectively.

**Figure 7:**
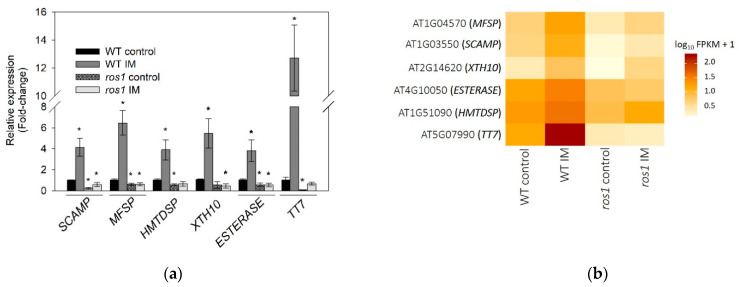
Transcriptionally regulated genes in response to herbicide application in wild type (WT) *A. thaliana* are not upregulated in the *ros1* mutant. (**a**) Quantitative PCR validation (fold-change) of genes from the differentially expressed genes (DEGs) profiling. Error bars indicate the standard deviation of three biological replicates and asterisks indicate significant differences between the treatments and *A. thaliana* wild type (WT) control according to *t*-test (*p* < 0.05); * compared to WT control. (**b**) Differentially expressed genes (DEGs) were performed by using CummeRbund, comparing genes in response to imazethapyr (IM) in *A. thaliana* wild type (WT) and *ros1*, the gene-normalized signal intensities are shown in the heat map.

Additionally, we performed GO classification and enrichment with the group of 89 genes that were induced by imazethapyr in the wild type and downregulated in *ros1* mutants (Figure S9). Detailed information of the biological process showed that the profile of these genes is overrepresented in a total of 22 pathways (Figure 6). The ontology of the 89 genes was analyzed (Table S4) and 31 candidate genes were identified as putative involved in herbicide detoxification (NTSR resistance), including two genes *P450*, three *GSTs*, 13 transporters, one oxidase, five glycosyl-transferase, and six esterases/hydrolases (Table 1). These genes were selected based on high variation in expression through imazethapyr application in the wild type and low expression in *ros1*. To confirm RNA-Seq data, six genes presented in Table S5 were selected for RT-qPCR analyses, which confirmed the RNA-seq expression pattern (Figure 7).

We performed in silico analysis of DNA methylation status of the 31 candidate genes potentially involved in NTSR using publicly available Epigenomics Data from the Jacobsen lab (http://genomes.mcdb.ucla.edu/AthBSseq/, accessed on 8 January 2021) (Table 1). The transcription start site upstream region of each gene was analyzed for the presence of transposable elements (TE) and/or CG, CHG, and CHH DNA methylation (where H is A, T or C) in wild type and *ROS1*, *DEMETER-LIKE PROTEIN 2* and *DEMETER-LIKE PROTEIN 3* (*rdd*) triple mutant (Table 1). Some of the analyzed genes indeed contained TE and/or DNA methylation in their promoter (Table 1), which may be an indication that some of these genes are regulated by the TGS.

**Figure 8:**
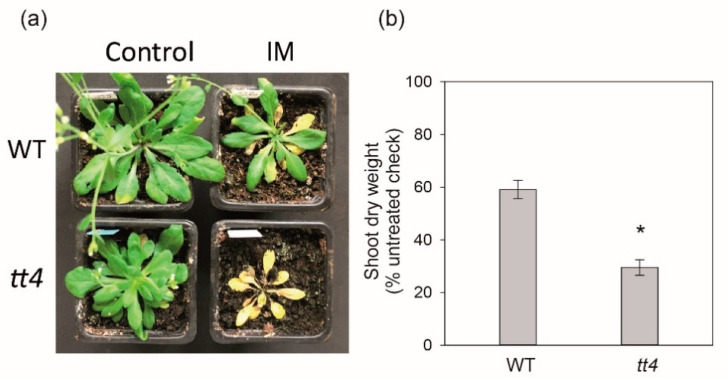
TT4 function for the rate-limiting step into flavonol production is necessary for *A. thaliana* tolerance to low herbicide dose. (**a**) Visual effect, (**b**) shoot dry weight of *A. thaliana* wild type (WT) and *tt4* mutant 14 days after application of imazethapyr (IM). Error bars indicate the standard deviation of four biological replicates, statistically significant differences relative to WT is indicated by asterisk (*t*-test, *p* ≤ 0.05); * compared to WT.

The metabolic processes most significantly enriched pathways of the 89 candidate genes were mainly involved with the flavonoid biosynthetic process and flavonoid metabolic process (Figures S10 and S11). To experimentally test for the role of flavonoids in the response to imazethapyr, *tt4* plants deficient in flavonoid-biosynthesis were treated with 10.6 g ha^−1^ imazethapyr (Figure 8). The visual estimate of the imazethapyr effect on *tt4* plants revealed more tissue damage as indicated by extensive loss of green color, leaf drying, and finally death (Figure 8a). Additionally, imazethapyr-treated *tt4* plants had approximately 30% lower shoot dry weight 14 days after imazethapyr treatment compared to the treated wild type (Figure 8b). Although the *TT4* is downregulated in *ros1* (Table S4), DNA methylation at the *TT4* locus in *ros1* presents no changes (Table 1), indicating that an unidentified direct target of *ROS1* is upstream of *TT4*. On the other hand, *TT7* is a candidate for epigenetic regulation as the promoter region is hypermethylated in *ros1* (Table 1). This suggests a positive correlation between flavonoid accumulation and imazethapyr stress response and may indicate that some genes of this pathway are epigenetically regulated.

## 3. Discussion

The identification of the appropriate herbicides sublethal doses was the first step in this study. The effect of herbicide sublethal dose simulates field situations where some plants receive a reduced rate of herbicide caused by failures in the application, plant canopy protection, and/or herbicide drift. These plants will suffer the herbicide effect to a smaller extent and may not die. In the scenario where herbicides induce intense stress in plants, acting similarly to other abiotic environmental stresses, weeds probably respond to herbicide application by activating stress-signaling networks and reprogramming gene expression [16,18,39]. An alternative scenario may be that there is random variation in the epigenome that affects expression from an herbicide resistance gene, which gets naturally selected and promoted by the herbicide application [40]. Thus, it is suggested that epigenetic mechanisms could be contributing to the ‘flipped on’ or ‘flipped off’ state of some specific genes related to herbicide resistance [17,19].

Gene silencing in plants can occur in transcriptional and post-transcriptional context. Methylation of a gene (or transgene) promoter typically correlates with TGS/RdDM [23,24]. We showed that exposures to sublethal doses of imazethapyr, glyphosate and 2,4-D lead to transient changes in TGS in *A. thaliana*. Using TGS reporter line L5, we showed that herbicides cause a minor loss of TGS and most L5 activation occurred in cotyledons. The intensity and localization of inhibitor-induced TGS suppression resemble that of lower concentrations of chemical inhibitors of DNA methylation including 5-azacytidine and zebularine [34,41]. This suggests that herbicides destabilize epigenome easier in tissues with only the maintenance TGS pathway being active. Besides, our candidate mutant screen indicated that active DNA demethylation by ROS1 pathway may be biologically more relevant in course of herbicide response in Arabidopsis.

In general, loss of TGS from pathways involved in DNA methylation, RNA interference and/or heterochromatic histone modifications leads to increased susceptibility to glyphosate, imazethapyr and 2,4-D. However, the response of each mutant was herbicide specific. Here, *ros1* plants were unique as they were hypersensitive to all tested herbicides. The differences found for the effect of different epigenetic pathways and herbicide are probably because the herbicides glyphosate, imazethapyr and 2,4-D belong to different modes of action and probably require different detoxification genes, plant transport or other metabolizing processes. Thus, these pathways may regulate different key genes involved in response to individual herbicides. For example, imazethapyr is a potent inhibitor of ALS, thereby stopping the synthesis of the branched-chain amino acids valine, leucine, and isoleucine, with subsequent plant death [42]. While glyphosate is a specific inhibitor of the chloroplast enzyme EPSPS [43] and 2,4-D is a synthetic auxin that produces uncontrolled and lethal growth in target plants [44].

ROS1 plays an important role in DNA demethylation and responses to developmental and environmental cues [45]. The control by both DNA methylation and demethylation is important for keeping the plant epigenome plasticity in response to ever-changing conditions [45]. ROS1 was proposed to function during salt stress conditions in tobacco [46]. In *Arabidopsis*, ROS1 is important for avoiding DNA hypermethylation at thousands of specific genomic loci [47]. Here, we show that ROS1 may influence the transcription of genes important to herbicide tolerance, induced by stimulus perception to adaptive plant behavior. The most likely mechanism may involve transcriptional control of specific detoxification of genes by ROS1. As hypomorphic mutant allele of *ros1* presented a strong increase in imazethapyr susceptibility, we utilized this herbicide to verify its effects on DNA methylation and transcriptome patterns in the wild type and *ros1* plants.

We showed a possible connection between the pattern of genomic DNA methylation and the alteration on susceptibility to imazethapyr because wild type treated with imazethapyr presented a lower level of 5mdC compared to *ros1*. Therefore, *ros1* plants may have genes important for herbicide detoxification under methylation control that are not able to be demethylated in the absence of ROS1. It suggests that ROS1 may be important for the detoxification of sublethal doses of the tested herbicides. Although HPLC analysis allowed to verify the different overall level of DNA methylation between the wild type treated with imazethapyr and *ros1*, this analysis does not permit to verify any DNA sequence sites, as well as genome location where this occurs. In this context, the next step for understanding the epigenetic mechanisms involved with the regulation of genes important for herbicide detoxification was to verify which genes were differentially expressed in the wild type and *ros1* after imazethapyr application.

Despite the accumulating transcriptomic profiles, limited information is available for the effect of herbicides on plant transcriptome [48,49]. Even more so concerning epigenetic mechanisms and their involvement with herbicide tolerance or resistance [18]. Our results showed that a sublethal dose of imazethapyr increased the expression of genes mainly linked to response to chemical stimulus, secondary metabolism, stress condition, and genes involved with flavonoid biosynthesis. This is congruent with biochemical assays results that indicated more anthocyanin and reactive oxygen species (ROS) were produced and photosynthetic activity was substantially decreased after 20 μg/L imazethapyr application in *A. thaliana* [48]. Additionally, our results showed that imazethapyr decreases the expression of genes involved in cell division and photosynthesis. This collaborates to affirm the common observation that ALS inhibitors have a very rapid and potent inhibition of cell division, with the result that inhibition of elongation of young roots and leaves is evident within 3 h after application [50].

A transcriptome study indicated that ALS-inhibitor response pathways are strongly conserved in plants *A. thaliana*, *Alopecurus myosuroides* (*Am*) and *Lolium* sp. The authors suggested that *A. thaliana* model could be used as a first step to discover the genes involved in ALS-inhibitor response pathways [51]. The identified genes putatively controlled by ROS1 in response to imazethapyr, included two cytP450, three GSTs, 13 transporters, one oxidase, five glycosyl-transferase, and six esterases/hydrolases, and products of these genes are mainly involved with the flavonoid biosynthetic and metabolic processes. The accumulation of flavonoids in plants is induced under the influence of abiotic stresses such as nitrogen, light, temperature, UV, and drought [52]. In grasses, studies have revealed that multiple herbicide resistance is connected to changes in endogenous antioxidant and secondary metabolism, particularly an accumulation of cytoprotectants such as glutathione, flavonoids and anthocyanins [53]. Arabidopsis transformed with *AmGSTF1* conferred multiple resistance and exerted a direct regulatory control on metabolism that led to an accumulation of protective flavonoids [54].

Here we showed that a flavonoid-deficient mutant (*tt4*) showed an increase in imazethapyr susceptibility contrasting with wild-type plants. TT4 is a CHALCONE SYNTHASE and acts at the first step of flavonoid biosynthesis, to produce naringenin chalcone. The remaining steps of flavonol production are further catalyzed by TT5, TT6, TT7, and FLAVONOL SYNTHASE [55,56]. The role of flavonoids in mediating herbicide tolerance was investigated in the flavonoid disabled *Arabidopsis* mutant *tt4* with the plants shown to be hypersensitive to imazethapyr treatment. These data suggest that flavonoid accumulation has also an effect on how plants respond to imazethapyr stress in *A. thaliana* and that some genes of this pathway are epigenetically regulated. These results indicate that DNA methylation is a potential additional adaptive mechanism for sublethal doses of imazethapyr.

To enable plant survival to herbicides, rapid and efficient mechanisms must allow herbicide neutralization [16,57]. Safeners have been predominantly used in the field to prevent crop injury without significantly reducing weed control [58]. Safeners associated with ALS inhibitors reduce the sensitivity of *Lolium* sp. plants to their associated herbicides by intensifying already existing NTSR pathways, by enhancing the effect on the expression level of 10 genes in *Lolium* sp. plants [59]. The study suggested a possible, uninvestigated way to NTSR evolution could be a selection for amplified responsiveness to safener action [59]. Our study focused on the ‘tolerance’ to herbicide treatments, and we show that various Arabidopsis mutants defective in different components of epigenetic mechanisms show that mutants impaired in DNA methylation are more susceptible to tested herbicides. From this point of view, if upregulation of various stress response genes can protect a plant from low herbicide dose, it stands to reason that further upregulation and/or upregulation of other genes may protect plants from higher doses, which is the basis of evolved resistance and how herbicide safeners work in crops. This is an interesting and potentially important area of research because it has the potential to change the understanding of the weed response to herbicides and the evolution of herbicide resistance.

Our results indicate that the evaluated herbicides can change specific epigenetic pathways according to the herbicide and suggest regulation of specific genes. Additionally, the active DNA demethylation process may be important for *A. thaliana* response to the herbicides glyphosate, 2,4-D, and especially to imazethapyr. These results together suggest that imazethapyr-induced changes in DNA methylation marks are possibly involved in an epigenetic mechanism associated with activation of specific genes responsive to imazethapyr. Additionally, ROS1 presents importance to the demethylation process induced by the herbicides and putative genes involved in response to herbicide stress are under the regulation of TGS machinery.

## 4. Materials and Methods

### 4.1. Plant Materials and Growth Conditions

All experiments were performed with *A. thaliana* accession Columbia-0 (Col-0). Furthermore, we used L5 reporter line carrying a single locus multicopy insertion of *Cauliflower Mosaic Virus 35S Promoter* fused to *β-Glucuronidase* coding sequence (*Pro35S::GUS*) gene, which is suppressed by TGS [25,32,33]. Plants double homozygous for L5 locus and mutation in *DDM1* chromatin remodeling factor (L5 *ddm1-5*) were used as low methylation control [34]. Eleven chromatin mutants associated with various TGS pathways were analyzed in this study, the description of mutants is according to TAIR (https://www.arabidopsis.org/, accessed on 10 February 2021): *upf1-5* (*UP-FRAMESHIFT;* SALK_112922); *ago6 (ARGONAUTE 6;* SALK_031553); *nrpe1* (*NUCLEAR RNA POL V, DEFECTIVE IN RNA-DIRECTED DNA METHYLATION; EMS mutant*); *nrpd2a* (*NUCLEAR RNA POLYMERASES IV AND V; DEFECTIVE IN RNA-DIRECTED DNA METHYLA-TION 2;* SALK_046208); *rdr6* (EMS mutant)*; ddm1-5* (82 bp insertion in exon 2)*; fas1* (SAIL_662_D10); *rpa2* (*REPLICATION PROTEIN A*; SALK_111834)*; ibm1* (SALK_035608)*; rts1-1* (37 bp deletion 21 bp downstream of the start codon); *ros1* (SALK_064264 hypomorphic allele). It has to be noted that the SALK_064264 mutant allele of *ros1* carries T-DNA insertion in the 5′-UTR. Based on the result of our RNA-seq data, this allele has 4-fold reduced amount of *ROS1* transcript (FPKM: WT = 16.6819 versus *ros1* = 4.07911; q-value = 0.000133). Hence the used *ros1* allele is a hypomorphic knock-down mutant.

Seeds were placed on moist soil, stratified for 48 h at 4 °C in dark, and then grown in a growth chamber (Percival) at 21 °C during the day and 16 °C during the night, relative humidity 70–75% and 150 µmol m^−2^ s^−1^ light intensity (16 h light/8 h dark). Three week old plants (approximately) were used for herbicide treatments.

### 4.2. Herbicide Treatments

The herbicides were applied using an automatic spray chamber (Greenhouse Spray Chamber; Generation III), with the TJ8002E spray nozzle, constant pressure of 289.6 lb pol^−2^ and velocity of 1.16 m s^−1^, providing a spray volume of 200 L ha^−1^. A dose–response experiment was conducted with wild type to determine the sublethal dose of the herbicides that were used for all subsequent experiments. The methodology of the dose–response curve was organized in a completely randomized design, with three replicates. Three herbicides of a different mode of action were used. Each herbicide was evaluated at 0%, 2.5%, 5%, 10%, and 20% of the label dose. The doses used were 0, 18, 36, 72, 144 g ha^−1^ for glyphosate (Roundup Original, 480 g/L CS, Monsanto S/A), 0, 2.6, 5.3, 10.6, 21.28 g ha^−1^ in addition of 0.5% *v*/*v* Dash for imazethapyr (Imazethapyr plus Nortox, 106 g/L CS, Nortox S/A) and 0, 20.1, 40.3, 80.6, 161.2 g ha^−1^ for 2,4-D (DMA 806 BR, 806 g/L CS, Dow AgroSciences S/A).

The sublethal dose for each herbicide was evaluated as plant injury and shoot dry weight at 20 DAT sublethal dose was considered the maximum dose that caused plant injury and biomass reduction but did not cause plant death. Plant injury was assessed visually using a scale from 0% (no injury) to 100% (plant death). Control ratings were based on symptoms of plants corresponding to each herbicide compared to non-treated control plants. Shoot dry weight was obtained by harvesting the plants and drying in an oven at 60 °C until constant weight. Data were tested for normality using PROC UNIVARIATE and subjected to analysis of variance (ANOVA) (*p* ≤ 0.05) in SAS. To satisfy the ANOVA premise for normality, plant injury and dry weight, data were transformed with X = 10 + arcsen√X100 and X = 1/X, respectively. After that, complementary regression analysis was performed, fitted to a nonlinear logistic model with three parameters [y = a/1 + (x/x0)^b^], as proposed by Streibig [60]. The determination of 50% plant injury and 50% growth reduction (GR50) was obtained by replacing “y” in the equation with 50. According to this experiment, the sublethal dose of each herbicide was established.

After determining the sublethal dose for each herbicide, the herbicide effect on TGS was evaluated. The experiment was conducted as a completely randomized design with three biological replicates. Sublethal doses of glyphosate, imazethapyr and 2,4-D were applied on 21 days old plants, the treated and non-treated plants were collected 48 h after herbicides application. The relative abundance of *GUS* transcript was measured by reverse transcription-quantitative PCR (RT-qPCR) and GUS histochemical staining was performed as previously described in Pecinka et al. [25]. Data sets were submitted to the *t*-test (*p* ≤ 0.05).

To determine the herbicide effect on *A. thaliana* chromatin mutants, the experiment was conducted twice (studies 1 and 2) and was organized in a completely randomized design, with three biological replicates. The evaluations of leaf length and shoot dry weight were performed 10 DAT. The leaf length measurement was based on pictures of the plant shoot followed by analysis with the ImageJ software. For each plant, the length of the fifth to the ninth leaf was analyzed. The measurement of dry weight was performed as described in the first experiment. Data were tested for normality using PROC UNIVARIATE and subjected to ANOVA (*p* ≤ 0.05) in SAS. To satisfy the ANOVA premise for normality, leaf length data were transformed with X = √X + 0.5. If statistical significance was found, the means were compared by the Tukey-test (*p* ≤ 0.05).

### 4.3. Global DNA Methylation Analysis by Isocratic Cation-Exchange High-Pressure Liquid Chromatography (HPLC)

The experiment was organized in a completely randomized design, with three biological replicates. Treated and non-treated plants were collected 48 h after herbicide application. DNA isolation was performed by using Nucleon PhytoPure gDNA Kit (GE Healthcare, Munich, Germany) according to the manufacturer’s instructions. DNA concentrations were quantified with the NanoDrop ND-1000 spectral photometer (peqLab, Erlangen, Germany). Treatment with RNase A (Thermo Fischer, Langenselbold, Germany) for 20 min at 37 °C was performed for all samples. The *ddm1* epigenetic mutant line and lambda phage DNA (N3011S; New England Biolabs, Frankfurt, Germany) were used as experiment controls (low methylation controls) [61]. A total of 1 µg genomic DNA per sample was used for analysis. Global 5-mdC levels of samples were analyzed as a percentage of 5-mdC relative to total deoxycytidine (dC) levels using cation-exchange HPLC [62]. The 5-mdC values were expressed as a percent of total cytosine. Data were tested for normality using PROC UNIVARIATE in SAS and statistically evaluated by ANOVA (*p* ≤ 0.05). If statistical significance was found, means were compared by the Tukey-test (*p* ≤ 0.05).

### 4.4. High-Throughput mRNA Sequencing (RNA-Seq)

RNA-seq analysis was performed in *A. thaliana* Col-0 wild type and *ros1* mutant. Treated and non-treated plants were collected 48 h after imazethapyr application. Each sample consisted of the entire rosette of two plants that were collected and immediately frozen in liquid nitrogen. Total RNA was isolated using the RNeasy Plant Mini Kit (Qiagen, Hilden, Germany) according to the manufacture’s protocol and with an additional on-column DNase I digestion (Roche, Basel, Switzerland). RNA concentrations were quantified by spectrophotometry using the Qubit RNA HS Assay kit and the Qubit Fluorometer (Life Technologies, Karlsruhe, Germany). RNA Integrity Numbers (RIN) were determined on a Bioanalyzer assay using the Agilent RNA 6000 Nano Kit (Böblingen, Germany). Samples with a RIN >8.5 were used for library construction. RNA-seq libraries were made using Illumina TruSeq RNA Sample Prep Kit (San Diego, CA, USA) following the manufacturer’s instructions. Subsequently, library concentrations were measured with the Qubit dsDNA HS Assay Kit on the Qubit Fluorometer and its insert size and integrity analyzed on a Bioanalyzer using the Agilent DNA 1000 Kit (Böblingen, Germany). High throughput sequencing was performed on an Illumina HiSeq2500 sequencer with a requested sequencing depth of 18.7 million 100-bp single-end reads per library at the Max Planck Genome Center (Cologne, Germany).

RNA-seq raw reads were quality controlled using FASTQC (Version 0.10.1) and low-quality bases were trimmed with the FASTX-toolkit [63] using standard parameters. The protocol used for differential gene and transcript expression analysis of RNA-seq was described by Trapnell et al. [64]. The libraries with sufficient quality were mapped to the corresponding reference genome *A. thaliana* TAIR10 [65] using bowtie2 and TopHat2 with default parameters [64,66]. The obtained files were indexed and visualized with CummeRbund (performed in the statistical software ‘R’) to facilitate exploration of genes identified by Cuffdiff as differentially expressed genes.

### 4.5. Gene Ontology (GO) Analysis of DEGs

To determine the functions of genes of interest, the gene ontology (GO) analysis of DEGs was performed. DEGs were assigned to different functional categories using PANTHER Classification System [67]. The annotations were verified manually and integrated using GO classification in three categories: biological process, molecular function, and cellular component. The detailed information of biological processes was performed using singular enrichment analysis (SEA) in agriGO.

### 4.6. Transcript Abundance Analysis by RT-qPCR

For RT-qPCR analysis, the entire plant rosette was collected and immediately frozen in liquid nitrogen. RNA extraction was performed using the RNeasy Plant Mini Kit (Qiagen, Hilden, Germany) according to the manufacturer’s protocol with an additional on-column DNase I digestion (Roche, Basel, Switzerland). RNA concentrations were quantified with the NanoDrop ND-1000 spectral photometer (peqLab, Erlangen, Germany). An amount of 1 µg of total RNA was reverse transcribed into complementary DNA (cDNA) with the First Strand cDNA Synthesis Kit using oligo(dT) primers (Thermo Scientific, St. LeonRot, Germany) according to the manufacturer’s protocol and analyzed by RT-qPCR with the SensiMix SYBR and Fluorescein Kit (Bioline, Berlin, Germany). Each reaction was set up in a 12 μL total volume, which contained 6 μL of 2x SensiMix SYBR and Fluorescein, 1 μL of primers (in a final concentration of 250 nM for each primer), 1 μL of water, and 4 μL of the cDNA template. The cDNA samples were diluted at a cDNA:distilled water ratio of 1:100. The reactions were carried out using the following cycling parameters: 95 °C for 5 min, followed by 40 cycles of 94 °C for 15 s, 60 °C for 10 s, 72 °C for 15 s, and 60 °C for 35 s. Primer sets used are shown in Table S5.

The RT-qPCR analysis was performed in biological triplicates, each with three technical replicates. The evaluation of RT-qPCR data was performed according to the MIQE (Minimum Information for Publication of Quantitative real-time PCR Experiments) [68]. After running the RT-qPCR, the melting curve of the PCR products was analyzed to control the homogeneity of the amplification product, where a sharp, narrow peak was required. Expression values were calculated relative to control-treated samples using the standard curve method [69] normalized to the herbicide stable reference genes *GLYCERALDEHYDE-3-PHOSPHATE DEHYDROGENASE C-2* (*GAPC-2*; AT1G13440) and *UBIQUITIN-CONJUGATING ENZYME 28* (*UBC28*; AT1G64230). Data sets were submitted to the *t*-test (*p* ≤ 0.05).

### 4.7. Accession Numbers

The following gene names and symbols are associated with this publication: *AGO6* (AT2G32940), *DDM1* (AT5G66750), *FAS1* (AT1G65470), *IBM1* (AT3G07610), *NRPD2A* (AT3G23780), *NRPE1* (AT2G40030), *RDR6* (AT3G49500), *ROS1* (AT2G36490), *RPA2* (AT2G24490), *RTS1* (AT5G63110), *TT4* (AT5G13930), *TT5* (AT3G55120), *TT7* (AT5G07990), *UPF1* (AT5G47010).

## Figures and Tables

**Table 1:** In silico analysis of ≈1 kb region upstream of the transcription start site of putative herbicide resistance genes (based on the DNA methylation browser by Jacobsen lab, UCLA; http://genomes.mcdb.ucla.edu/AthBSseq/, accessed on 8 January 2021).

Upstream Region/Presence of:					
Putative Herbicide Resistance Genes			TE ^†^	5mdC ^‡^	*rdd* Change ^§^
Cytochromes P450					
1	AT4G19230	*CYTOCHROME P450, FAMILY 707, SUBFAMILY A, POLYPEPTIDE 1 (CYP707A1);*	yes	no	no
2	AT1G19630	*CYTOCHROME P450, FAMILY 722, SUBFAMILY A, POLYPEPTIDE 1 (CYP722A1);*	no	no	no
3	AT5G07990	*TRANSPARENT TESTA 7 (TT7); OR CYTOCHROME P450 75B1*	no	yes	yes
GST					
4	AT2G29490	*GLUTATHIONE S-TRANSFERASE TAU 1 (GSTU1);*	no	no	no
5	AT5G17220	*GLUTATHIONE S-TRANSFERASE PHI 12 (GSTF12);*	no	no	no
6	AT1G17170	*GLUTATHIONE S-TRANSFERASE TAU 24 (GSTU24);*	no	yes	no
Transporters					
7	AT2G04070	*MATE EFFLUX FAMILY PROTEIN;*	no	no	no
8	AT1G51090	*HEAVY METAL TRANSPORT/DETOXIFICATION SUPERFAMILY PROTEIN;*	no	yes	yes
9	AT1G43890	*RAB GTPASE HOMOLOG B18 (RAB18);*	no	yes	yes
10	AT4G21910	*MATE EFFLUX FAMILY PROTEIN;*	yes	no	no
11	AT4G35060	*HEAVY METAL TRANSPORT/DETOXIFICATION SUPERFAMILY PROTEIN;*	no	no	no
12	AT1G70300	*K+ UPTAKE PERMEASE 6 (KUP6);*	yes	no	no
13	AT5G47560	*TONOPLAST DICARBOXYLATE TRANSPORTER (TDT);*	no	no	no
14	AT1G09180	*SECRETION-ASSOCIATED RAS SUPER FAMILY 1 (SARA1A);*	no	no	no
15	AT1G03550	*SECRETORY CARRIER MEMBRANE PROTEIN (SCAMP) FAMILY PROTEIN;*	yes	yes	yes
16	AT1G31820	*AMINO ACID PERMEASE FAMILY PROTEIN;*	no	no	no
17	AT1G04570	*MAJOR FACILITATOR SUPERFAMILY PROTEIN;*	yes	yes	yes
18	AT2G41190	*TRANSMEMBRANE AMINO ACID TRANSPORTER FAMILY PROTEIN;*	no	no	no
19	AT3G46450	*SEC14 CYTOSOLIC FACTOR FAMILY PROTEIN/ PHOSPHOGLYCERIDE TRANSFER FAMILY PROTEIN;*	no	no	no
Oxidases					
20	AT4G20860	*FAD-BINDING BERBERINE FAMILY PROTEIN;*	no	no	no
Glycosyl-transferase					
21	AT2G43820	*UDP-GLUCOSYLTRANSFERASE 74F2 (UGT74F2);*	no	no	no
22	AT5G54060	*UDP-GLUCOSE: FLAVONOID* *3-O-GLUCOSYLTRANSFERASE (UF3GT);*	no	no	no
23	AT1G24070	*CELLULOSE SYNTHASE-LIKE A10 (CSLA10);*	no	no	no
24	AT1G56600	*GALACTINOL SYNTHASE 2 (GOLS2);*	no	no	no
25	AT1G05675	*UDP-GLYCOSYLTRANSFERASE SUPERFAMILY PROTEIN;*	no	no	no
Esterases/hydrolase					
26	AT4G10050	*ESTERASE/LIPASE/THIOESTERASE FAMILY PROTEIN;*	no	yes	yes
27	AT1G54020	*GDSL-LIKE LIPASE/ACYLHYDROLASE SUPERFAMILY PROTEIN;*	no	no	no
28	AT1G47510	*INOSITOL POLYPHOSPHATE 5-PHOSPHATASE 11 (5PTASE11);*	yes	no	no
29	AT3G43580	*BETA-GALACTOSIDASE RELATED PROTEIN;*	no	yes	yes
30	AT2G14620	*XYLOGLUCAN ENDOTRANSGLUCOSYLASE/* *HYDROLASE 10 (XTH10);*	yes	no	yes
31	AT5G50400	*PURPLE ACID PHOSPHATASE 27 (PAP27);*	yes	yes	yes

^†^ Presence of transposable element (TE); ^‡^ Presence of 5 methyl-2-deoxy-cytosine (5mdC) as known as DNA methylation in CH. CHG and CHH sequence contexts (where H is A, T or C); ^§^ DNA methylation change in *ros1 dml2 dml3* (*rdd*) triple DNA demethylase mutant.

## Data Availability

RNA sequencing reads are deposited in Gene Expression Omnibus as the study number GSE147225.

## Supplementary Materials

The following are available online at https://www.mdpi.com/1422-0067/22/7/3314/s1, Figure S1: The visual effect of *A. thaliana* Col-0 wild type (WT) 20 days after treatment (DAT) of glyphosate (a,b), imazethapyr (c,d), and 2,4-D (e,f). Yellow bars correspond to 7 cm. (b,d,f) Plant injury (%) of *A. thaliana* treated with glyphosate, imazethapyr and 2,4-D, respectively, at 20 DAT. The graphs were plotted with the average and the vertical bars indicate the confidence interval. Regression analysis was performed, fitted to a nonlinear logistic model with three parameters [y = a/1 + (x/x0)b], proposed by Streibig [60]; Figure S2: Shoot dry weight (g plant^−1^) of *A. thaliana* Col-0 wild type (WT) 20 days after treatment (DAT) of glyphosate (a), imazethapyr (b) and 2,4-D (c). The graphs were plotted with the average and the vertical bars indicate the confidence interval. Regression analysis was performed, fitted to a nonlinear logistic model with three parameters [y = a/1 + (x/x0)^b^], proposed by Streibig [60]; Figure S3: Leaf length (% untreated check) of *A. thaliana* Col-0 wild type (WT) and mutants, at 10 days after treatment (DAT) of glyphosate (a), imazethapyr (b) and 2,4-D (c). Means of studies one and two followed by different letters differ significantly according to Tukey’s test (*p* ≤ 0.05); ns = nonsignificant. Mutant followed by asterisk differ significantly from WT according to Tukey’s test (*p* ≤ 0.05); Figure S4. Bioinformatic analysis of RNA-seq data by using CummeRbund plots. (a) Expression level distribution for all genes in wild type (WT) and *ros1* mutant, in control (mock) condition and imazethapyr treatment (IM); FPKM, fragments per kilobase of transcript per million fragments mapped reads. (b) Volcano plots showing significant (adjusted *p*-value < 0.05) differentially expressed genes, in red color; Figure S6. Detailed information of biological process representing the percent of genes involved in biological process pathways of upregulated in wild type (WT) 48 h after imazethapyr (IM) treatment, performed by using singular enrichment analysis (SEA) in AgriGo. Gray and black bars indicate the percent of genes related to the input list and the percent of genes compared to genome reference, respectively; Figure S7. Pie chart representing Gene Ontology (GO) of downregulated genes in *A. thaliana* Col-0 wild type (WT) 48 h after imazethapyr (IM) treatment. A total of 1164 differential expression of genes (DEGs) were annotated in at least one of the three GO categories: cellular component, biological process, and molecular function; Figure S8. Detailed information of biological process representing the percent of genes involved in biological process pathways downregulated in wild type (WT) 48 h after imazethapyr (IM) treatment, performed by using singular enrichment analysis (SEA) in AgriGo. Gray and black bars indicate the percent of genes related to the input list and the percent of genes compared to genome reference, respectively; Figure S9. Pie chart representing Gene Ontology (GO) of 89 genes of interest induced by imazethapyr (IM) in *A. thaliana* wild type (WT) and downregulated in *ros1* mutant. A total of 75 differential expression of genes (DEGs) were annotated in at least one of the three GO categories: cellular component biological process and molecular function; Figure S10. Part I of the overview of pathways overrepresented, according to singular enrichment analysis (SEA). The color scale indicates the significance levels of enrichment analysis. The arrows represent the relationship between parent–child terms; Figure S9. Part II of the overview of pathways overrepresented, according to singular enrichment analysis (SEA). The color scale indicates the significance levels of enrichment analysis. The arrows represent the relationship between parent–child terms; Table S1. Parameters of the logistic equation of plant injury and shoot dry weight of *A. thaliana* Col-0 wild type (WT) treated with glyphosate, imazethapyr and 2,4-D; Table S2. Summary of reads obtained by Tophat analysis based on the RNA-seq data. R = biological replicate; IM = imazethapyr; Table S3. Genes involved with chromatin mechanisms (TAIR10) significantly up and downregulated 48 h after imazethapyr (IM) treatment in *A. thaliana* wild type (WT), according to RNA-seq data; Table S4. List of 89 genes of interest induced by imazethapyr (IM) in *A. thaliana* wild type (WT) and downregulated in *ros1* mutant; Table S5. Primers used in the analysis of transcription levels of GUS and for quantitative RT-PCR validation of genes by RNA-Seq.
