# Supplementary material for: Insights into the Role of Transcriptional Gene Silencing in Response to Herbicide-Treatments in Arabidopsis thaliana
Source: Int J Mol Sci. 2021 Mar 24;22(7):3314. doi: 10.3390/ijms22073314 (PMC8037345; doi:10.3390/ijms22073314)
Supplement: Supplementary file 1 [file ijms-22-03314-s001.pdf]

Supplementary Materials

RESULTS

Table S1. Parameters of the logistic equation of plant injury and shoot dry weight of *A. thaliana* Col-0 wild type (WT) treated with glyphosate, imazethapyr and 2,4-D.

Treatment	[1]a	[2]b	[3]X_0	[4]R^2	[5]PI_{50} (g ha^{-1})
			**Plant injury**		
Glyphosate	77.76**	-1.72*	27.78**	0.99	39.11
Imazethapyr	97.04**	-1.67*	4.27*	0.98	4.43
2,4-D	87.95**	-3.54*	22.89**	0.99	24.74
Treatment	[1]a	[2]b	[3]X_0	[4]R^2	[6]GR_{50} (g ha^{-1})
			**Shoot dry weight**		
Glyphosate	1.07**	1.49*	31.47*	0.99	31.31
Imazethapyr	1.02**	1.28*	4.07**	0.99	4.07
2,4-D	0.79**	1.45*	27.05*	0.98	27.05

[1]a: the difference between the maximum and minimum points of the curve;
[2]b: the slope of the curve;
[3]X_0: the dose which provides 50% of the response of the variable;
[4]R^2: coefficient of determination;
[5]PI_{50}: dose required to provide 50% plant injury, obtained by replacing "y" of the equation with 50;
[6]GR_{50}: dose required to reduce shoot weight by 50%; obtained by replacing "y" of the equation with 50% of the shoot dry weight;
** p<0.01; *p<0.05; NS non-significant;

Table S2. Summary of reads obtained by Tophat analysis based on the RNA-seq data. R = biological replicate; IM = imazethapyr.

Sample	Total Reads	Mapped Reads	Uniquely Mapped Reads	Unmapped Reads	Non-uniquely Mapped Reads
WT control – R1	20355943	19608630	18790352	747313	1565591
		**96.33%**	**92.31%**	**3.67%**	**7.69%**
WT control – R2	22575110	22049429	20941997	525681	1633113
		**97.67%**	**92.77%**	**2.33%**	**7.23%**
WT IM – R1	20502313	19819046	19027463	683267	1474850
		**96.67%**	**92.81%**	**3.33%**	**7.19%**
WT IM – R1	20286614	19552987	18758219	733627	1528395
		**96.38%**	**92.47%**	**3.62%**	**7.53%**
ros1 control – R1	20342194	18001244	17262400	2340950	3079794
		**88.49%**	**84.86%**	**11.51%**	**15.14%**
ros1 control – R2	17373574	14891533	14282008	2482041	3091566
		**85.71%**	**82.21%**	**14.29%**	**17.79%**
ros1 IM – R1	20012495	17719864	16998840	2292631	3013655
		**88.54%**	**84.94%**	**11.46%**	**15.06%**
ros1 IM – R2	21765920	19080090	18326741	2685830	3439179
		**87.66%**	**84.20%**	**12.34%**	**15.80%**
TOTAL:	**163.214.163**				

Table S3. Genes involved with chromatin mechanisms (TAIR10) significantly up and down-regulated 48 hours after imazethapyr (IM) treatment in *A. thaliana* wild type (WT), according to RNA-seq data.

Gene ID	Gene Annotation	Control	IM	Log2 Fold Change	Adjusted P Value‡
		FPKM†			
**Up-regulated genes**					
AT2G21450	*CHROMATIN REMODELING 34 (CHR34)*	0.08	5.95	6.15	0.00215
AT2G39030	*ACYL-COA N-ACYLTRANSFERASES (NAT) SUPERFAMILY PROTEIN*	5.19	148.67	4.84	5.00E-05
AT2G18050	*HISTONE H1-3 (HIS1-3)*	8.72	202.63	4.54	5.00E-05
AT2G32020	*ACYL-COA N-ACYLTRANSFERASES (NAT) SUPERFAMILY PROTEIN*	0.53	7.70	3.87	0.00045
AT1G31290	*ARGONAUTE 3 (AGO3)*	0.20	2.20	3.49	5.00E-05
AT2G32030	*ACYL-COA N-ACYLTRANSFERASES (NAT) SUPERFAMILY PROTEIN*	0.81	5.06	2.64	5.00E-05
AT1G64490	*DEK, CHROMATIN ASSOCIATED PROTEIN*	13.20	47.99	1.86	5.00E-05
AT1G13370	*HISTONE SUPERFAMILY PROTEIN*	1.12	3.46	1.63	0.005
AT5G42060	*DEK, CHROMATIN ASSOCIATED PROTEIN*	21.09	63.74	1.60	6.45371
AT1G05490	*CHROMATIN REMODELING 31 (CHR31)*	0.40	1.09	1.46	5.00E-05
AT1G02740	*MRG FAMILY PROTEIN; CHROMATIN ASSEMBLY OR DISASSEMBLY*	4.41	11.33	1.36	5.00E-05
AT5G20420	*CHROMATIN REMODELING 42 (CHR42)*	0.55	1.36	1.30	5.00E-05
AT3G44750	*HISTONE DEACETYLASE 3 (HDA3)*	55.69	135.64	1.28	5.00E-05
AT1G08460	*HISTONE DEACETYLASE 8 (HDA08)*	14.65	33.87	1.21	5.00E-05
AT1G03750	*SWITCH 2 (SWI2)*	2.47	5.61	1.18	3.48208
AT1G77540	*ACYL-COA N-ACYLTRANSFERASES (NAT) SUPERFAMILY PROTEIN*	26.68	56.79	1.09	4.38593
AT3G26850	*HISTONE-LYSINE N-METHYLTRANSFERASES*	6.32	13.02	1.04	0.0013

AT5G26040	*HISTONE DEACETYLASE 2 (HDA2)*	6.15	12.60	1.04	0.00965
AT1G18800	*NAP1-RELATED PROTEIN 2 (NRP2)*	26.31	51.29	0.96	5.00E-05
AT5G02560	*HISTONE H2A 12 (HTA12)*	17.17	33.40	0.96	5.00E-05
AT1G31280	*ARGONAUTE 2 (AGO2)*	7.50	14.33	0.93	5.00E-05
AT2G39000	*ACYL-COA N-ACYLTRANSFERASES (NAT) SUPERFAMILY PROTEIN*	20.53	38.64	0.91	5.00E-05
AT1G62310	*TRANSCRIPTION FACTOR JUMONJI (JMJC) DOMAIN-CONTAINING PROTEIN*	8.48	15.14	0.84	5.00E-05
AT2G44980	*SNF2 DOMAIN-CONTAINING PROTEIN/ HELICASE DOMAIN-CONTAINING PROTEIN*	3.64	6.49	0.84	5.00E-05
AT2G30280	*RNA-DIRECTED DNA METHYLATION 4 (RDM4)*	8.18	14.53	0.83	5.00E-05
AT4G17080	*HISTONE H3 K4-SPECIFIC METHYLTRANSFERASE SET7/9 FAMILY PROTEIN*	8.66	15.30	0.82	5.00E-05
AT1G03650	*ACYL-COA N-ACYLTRANSFERASES (NAT) SUPERFAMILY PROTEIN*	4.67	8.19	0.81	0.00195
AT3G51880	*HIGH MOBILITY GROUP B1 (HMGB1)*	89.36	155.70	0.80	5.00E-05
AT3G22680	*RNA-DIRECTED DNA METHYLATION 1 (RDM1)*	16.21	27.36	0.76	0.0001
AT2G27840	*HISTONE DEACETYLASE ACTIVITY HDT4*	32.81	54.97	0.74	5.00E-05
AT3G07610	*INCREASE IN BONSAI METHYLATION 1 (IBM1)*	10.69	17.79	0.73	5.00E-05
AT2G06990	*HUA ENHANCER 2 (HEN2)*	14.40	23.17	0.69	5.00E-05
AT5G14620	*DOMAINS REARRANGED METHYLTRANSFERASE 2 (DRM2)*	6.11	9.66	0.66	0.00015
AT2G38950	*TRANSCRIPTION FACTOR JUMONJI (JMJ) FAMILY PROTEIN*	17.57	27.18	0.63	5.00E-05
AT2G39020	*ACYL-COA N-ACYLTRANSFERASES (NAT) SUPERFAMILY PROTEIN*	56.00	77.09	0.46	0.00035
AT1G21920	*HISTONE H3 K4-SPECIFIC METHYLTRANSFERASE SET7/9 FAMILY PROTEIN*	8.86	11.94	0.43	0.00935

AT5G61060	*HISTONE DEACETYLASE 5 (HDA05)*	16.08	21.06	0.39	0.0029
AT2G39030	*RNA POLYMERASE II LARGE SUBUNIT (NRPB1)*	27.83	36.38	0.39	0.0012
AT3G18520	*HISTONE DEACETYLASE 15 (HDA15)*	17.94	22.28	0.31	0.0178
AT1G14790	*RNA-DEPENDENT RNA POLYMERASE 1 (RDR1)*	8.59	10.65	0.31	0.02225
**Down-regulated genes**					
AT5G44800	*CHROMATIN REMODELING 4 (CHR4)*	15.47	12.40	-0.32	0.0071
AT2G44150	*HISTONE-LYSINE N-METHYLTRANSFERASE ASHH3 (ASHH3)*	11.68	9.05	-0.37	0.0264
AT5G56740	*HISTONE ACETYLTRANSFERASE OF THE GNAT FAMILY 2 (HAG2)*	17.65	13.55	-0.38	0.0137
AT5G08430	*SWIB/MDM2 DOMAIN*	13.58	9.96	-0.45	0.00395
AT2G28720	*HISTONE SUPERFAMILY PROTEIN*	96.07	69.51	-0.47	0.0002
AT5G27670	*HISTONE H2A 7 (HTA7)*	120.74	87.28	-0.47	0.0001
AT4G29730	*NUCLEOSOME/CHROMATIN ASSEMBLY FACTOR GROUP C5 (NFC5)*	7.22	5.17	-0.48	0.00955
AT4G40030	*HISTONE SUPERFAMILY PROTEIN*	415.57	292.48	-0.51	5.00E-05
AT5G18620	*CHROMATIN REMODELING FACTOR17 (CHR17)*	18.97	13.29	-0.51	5.00E-05
AT4G13460	*SU(VAR)3-9 HOMOLOG 9 (SUVH9)*	23.67	16.09	-0.56	5.00E-05
AT2G36490	*DEMETER-LIKE 1 (DML1)*	25.00	16.68	-0.58	5.00E-05
AT5G13960	*SU(VAR)3-9 HOMOLOG 4 (SUVH4)*	7.64	5.06	-0.60	0.00105
AT2G27040	*ARGONAUTE 4 (AGO4)*	24.06	15.48	-0.64	5.00E-05
AT4G40030	*HISTONE SUPERFAMILY PROTEIN*	*415.57*	*292.48*	*-0.51*	*5.00E-05*
AT5G18620	*CHROMATIN REMODELING FACTOR17 (CHR17)*	18.97	13.29	-0.51	5.00E-05
AT4G13460	*SU(VAR)3-9 HOMOLOG 9 (SUVH9)*	23.67	16.09	-0.56	5.00E-05
AT2G36490	*DEMETER-LIKE 1 (DML1)*	25.00	16.68	-0.58	5.00E-05
AT5G13960	*SU(VAR)3-9 HOMOLOG 4 (SUVH4)*	7.64	5.06	-0.60	0.00105
AT2G27040	*ARGONAUTE 4 (AGO4)*	24.06	15.48	-0.64	5.00E-05
AT5G43990	*ZINC ION BINDING, HISTONE-LYSINE N-*	6.64	4.04	-0.72	0.00015

	METHYLTRANSFERASE ACTIVITY SUVR2				
AT5G63950	*CHROMATIN REMODELING 24 (CHR24)*	7.89	4.30	-0.88	5.00E-05
AT5G61070	*HISTONE DEACETYLASE OF THE RPD3/HDA1 SUPERFAMILY 18 (HDA18)*	1.16	0.63	-0.88	0.0135
AT3G27180	*S-ADENOSYL-L-METHIONINE-DEPENDENT METHYLTRANSFERASES SUPERFAMILY PROTEIN*	27.91	14.62	-0.93	5.00E-05
AT5G64150	*RNA METHYLTRANSFERASE FAMILY PROTEIN*	13.56	6.41	-1.08	5.00E-05
AT4G33470	*HISTONE DEACETYLASE 14 (HDA14)*	48.01	20.00	-1.26	5,00E-05
AT4G27230	*HISTONE H2A 2 (HTA2)*	84.29	35.01	-1.27	5.00E-05
AT5G64630	*FASCIATA 2 (FAS2)*	6.91	2.74	-1.33	5.00E-05
AT1G65470	*FASCIATA 1 (FAS1)*	9.50	3.71	-1.36	5.00E-05
AT5G66750	*CHROMATIN REMODELING 1 (CHR1)*	8.73	3.06	-1.51	5.00E-05
AT3G28460	*METHYLTRANSFERASES*	40.87	13.87	-1.56	5.00E-05
AT4G19020	*CHROMOMETHYLASE 2 (CMT2)*	10.60	3.31	-1.68	5.00E-05
AT1G51060	*HISTONE H2A 10 (HTA10)*	263.45	70.29	-1.91	5.00E-05
AT2G28740	*HISTONE H4 (HIS4)*	303.29	80.88	-1.91	5.00E-05
AT1G69770	*CHROMOMETHYLASE 3 (CMT3)*	19.41	4.95	-1.97	5.00E-05
AT3G20670	*HISTONE H2A 13 (HTA13)*	120.90	30.31	-2.00	5.00E-05
AT3G54560	*HISTONE H2A 11 (HTA11)*	76.58	16.47	-2.22	5.00E-05
AT3G46320	*HISTONE SUPERFAMILY PROTEIN*	165.57	34.32	-2.27	5.00E-05
AT5G59870	*HISTONE H2A 6 (HTA6)*	267.30	51.66	-2.37	5.00E-05
AT5G65350	*HISTONE 3 11 (HTR11)*	9.38	1.55	-2.60	5.00E-05

† Fragments per kilobase of transcript per million fragments mapped (FPKM) is an average of two biological replicates 48 hours after imazethapyr (IM) treatment;
‡ Adjusted P values were calculated using Cuffdiff statistics.

Table S4. List of 89 genes of interest induced by imazethapyr (IM) in *A. thaliana* wild type (WT) and down-regulated in *ros1* mutant.

	Gene ID	Gene description
1	AT1G51090	*HEAVY METAL TRANSPORT/DETOXIFICATION SUPERFAMILY PROTEIN;*
2	AT5G23410	*OTHER_RNA;*
3	AT1G66830	*LEUCINE-RICH REPEAT PROTEIN KINASE FAMILY PROTEIN;*

4	AT1G31835	*FUNCTIONS IN: MOLECULAR_FUNCTION UNKNOWN; INVOLVED IN: BIOLOGICAL_PROCESS UNKNOWN;*
5	AT5G42850	*THIOREDOXIN SUPERFAMILY PROTEIN;*
6	AT4G10050	*ESTERASE/LIPASE/THIOESTERASE FAMILY PROTEIN;*
7	AT1G54020	*GDSL-LIKE LIPASE/ACYLHYDROLASE SUPERFAMILY PROTEIN;*
8	AT4G13900	*PSEUDOGENE OF RECEPTOR LIKE PROTEIN 47;*
9	AT1G52347	*NONE*
10	AT5G03285	*OTHER_RNA;*
11	AT2G34655	*UNKNOWN PROTEIN;*
12	AT3G55290	*NAD(P)-BINDING ROSSMANN-FOLD SUPERFAMILY PROTEIN;*
13	AT1G24580	*RING/U-BOX SUPERFAMILY PROTEIN;*
14	AT2G39100	*RING/U-BOX SUPERFAMILY PROTEIN;*
15	AT4G14590	*EMBRYO DEFECTIVE 2739 (EMB2739);*
16	AT1G64180	*INTRACELLULAR PROTEIN TRANSPORT PROTEIN USO1-RELATED;*
17	AT5G42810	*INOSITOL-PENTAKISPHOSPHATE 2-KINASE 1 (IPK1);*
18	AT3G58350	*RESTRICTED TEV MOVEMENT 3 (RTM3);*
19	AT1G30160	*PROTEIN OF UNKNOWN FUNCTION (DUF295);*
20	AT5G43403	*OTHER_RNA;*
21	AT5G47590	*HEAT SHOCK PROTEIN HSP20/ALPHA CRYSTALLIN FAMILY;*
22	AT1G62570	*FLAVIN-MONOOXYGENASE GLUCOSINOLATE S-OXYGENASE 4 (FMO GS-OX4);*
23	AT2G43590	*CHITINASE FAMILY PROTEIN;*
24	AT2G17280	*PHOSPHOGLYCERATE MUTASE FAMILY PROTEIN;*
25	AT5G41960	*UNKNOWN PROTEIN;*
26	AT2G40110	*YIPPEE FAMILY PUTATIVE ZINC-BINDING PROTEIN;*
27	AT2G16367	*DEFENSIN-LIKE (DEFL) FAMILY PROTEIN.*
28	AT3G60520	*ZINC ION-BINDING PROTEIN;*
29	AT5G44578	*TRANSMEMBRANE PROTEIN;*
30	AT1G15580	*INDOLE-3-ACETIC ACID INDUCIBLE 5 (IAA5);*
31	AT5G43860	*CHLOROPHYLLASE 2 (CLH2);*
32	AT2G26215	*TRANSPOSABLE_ELEMENT_GENE;*
33	AT1G20490	*AMP-DEPENDENT SYNTHETASE AND LIGASE FAMILY PROTEIN;*
34	AT4G37580	*HOOKLESS 1 (HLS1);*
35	AT1G62540	*FLAVIN-MONOOXYGENASE GLUCOSINOLATE S-OXYGENASE 2 (FMO GS-OX2);*
36	AT3G24927	*PSEUDOGENE OF EXPRESSED PROTEIN;*
37	AT5G28622	*NON-LTR RETROTRANSPOSON FAMILY (LINE);*
38	AT5G42730	*PSEUDOGENE SIMILAR TO ACT DOMAIN-CONTAINING PROTEIN, SIMILAR TO F-BOX FAMILY PROTEIN*
39	AT2G38430	*UNKNOWN PROTEIN;*
40	AT4G14560	*INDOLE-3-ACETIC ACID INDUCIBLE (IAA1);*
41	AT3G47965	*UNKNOWN PROTEIN;*
42	AT5G48880	*PEROXISOMAL-3-KETO-ACYL-COA THIOLASE 1 (PKT1);*
43	AT2G42540	*COLD-REGULATED 15A (COR15A);*
44	AT1G73870	*B-BOX TYPE ZINC FINGER PROTEIN WITH CCT DOMAIN;*

45	AT5G50400	*PURPLE ACID PHOSPHATASE 27 (PAP27);*
46	AT2G33830	*DORMANCY/AUXIN ASSOCIATED FAMILY PROTEIN;*
47	AT3G13540	*MYB DOMAIN PROTEIN 5 (MYB5);*
48	AT1G15405	*OTHER_RNA;*
49	AT5G08600	*U3 RIBONUCLEOPROTEIN (UTP) FAMILY PROTEIN;*
50	AT5G13930	*TRANSPARENT TESTA 4 (TT4);*
51	AT5G38565	*F-BOX/FBD-LIKE DOMAINS CONTAINING PROTEIN;*
52	AT5G39090	*HXXXD-TYPE ACYL-TRANSFERASE FAMILY PROTEIN;*
53	AT5G05270	*CHALCONE-FLAVANONE ISOMERASE FAMILY PROTEIN;*
54	AT2G23910	*NAD(P)-BINDING ROSSMANN-FOLD SUPERFAMILY PROTEIN;*
55	AT5G24770	*VEGETATIVE STORAGE PROTEIN 2 (VSP2);*
56	AT3G28007	*NODULIN MTN3 FAMILY PROTEIN;*
57	AT2G46410	*CAPRICE (CPC);*
58	AT1G19630	*CYTOCHROME P450, FAMILY 722, SUBFAMILY A, POLYPEPTIDE 1 (CYP722A1);*
59	AT1G31820	*AMINO ACID PERMEASE FAMILY PROTEIN;*
60	AT5G52940	*PROTEIN OF UNKNOWN FUNCTION (DUF295);*
61	AT5G38895	*RING/U-BOX SUPERFAMILY PROTEIN;*
62	AT3G24780	*UNCHARACTERISED CONSERVED PROTEIN UCP015417;*
63	AT3G29590	*AT5MAT;*
64	AT2G37260	*TRANSPARENT TESTA GLABRA 2 (TTG2);*
65	AT3G42806	*TRANSPOSABLE_ELEMENT_GENE;*
66	AT2G23330.1	*TRANSPOSABLE_ELEMENT_GENE;*
67	AT1G53160	*SQUAMOSA PROMOTER BINDING PROTEIN-LIKE 4 (SPL4);*
68	AT3G24982	*RECEPTOR LIKE PROTEIN 40 (RLP40);*
69	AT1G24070	*CELLULOSE SYNTHASE-LIKE A10 (CSLA10);*
70	AT5G37440	*CHAPERONE DNAJ-DOMAIN SUPERFAMILY PROTEIN;*
71	AT4G01060	*CAPRICE-LIKE MYB3 (CPL3);*
72	AT1G30500	*NUCLEAR FACTOR Y, SUBUNIT A7 (NF-YA7);*
73	AT1G65500	*SECRETED PEPTIDE WHICH FUNCTIONS IN PLANT GROWTH AND PATHOGEN DEFENSE (STMP6);*
74	AT5G15960	*STRESS-INDUCED PROTEIN KIN1;*
75	AT5G61160	*ANTHOCYANIN 5-AROMATIC ACYLTRANSFERASE 1 (AACT1);*
76	AT5G54060	*UDP-GLUCOSE:FLAVONOID 3-O-GLUCOSYLTRANSFERASE (UF3GT);*
77	AT2G15128	*OTHER_RNA*
78	AT4G22880	*LEUCOANTHOCYANIDIN DIOXYGENASE (LDOX);*
79	AT1G56600	*GALACTINOL SYNTHASE 2 (GOLS2);*
80	AT5G17220	*GLUTATHIONE S-TRANSFERASE PHI 12 (GSTF12);*
81	AT4G09820	*TRANSPARENT TESTA 8 (TT8);*
82	AT5G60250	*ZINC FINGER (C3HC4-TYPE RING FINGER) FAMILY PROTEIN;*
83	AT1G58225	*UNKNOWN PROTEIN;*
84	AT5G07990	*TRANSPARENT TESTA 7 (TT7);*
85	AT5G42800	*DIHYDROFLAVONOL 4-REDUCTASE (DFR);*
86	AT1G14520	*MYO-INOSITOL OXYGENASE 1 (MIOX1);*
87	AT1G76960	*UNKNOWN PROTEIN;*

88	AT4G01985	*UNKNOWN PROTEIN;*
89	AT2G04070	*MATE EFFLUX FAMILY PROTEIN;*

Figure S1. The visual effect of *A. thaliana* Col-0 wild type (WT) 20 days after treatment (DAT) of glyphosate (a and b), imazethapyr (c and d), and 2,4-D (e and f). Yellow bars correspond to 7 cm. (b, d and f) Plant injury (%) of *A. thaliana* treated with glyphosate, imazethapyr and 2,4-D, respectively, at 20 DAT. The graphs were plotted with the average and the vertical bars indicate the confidence interval. Regression analysis was performed, fitted to a non-linear logistic model with three parameters [y= a/1+(x/x0)b], proposed by Streibig (1988) [60].

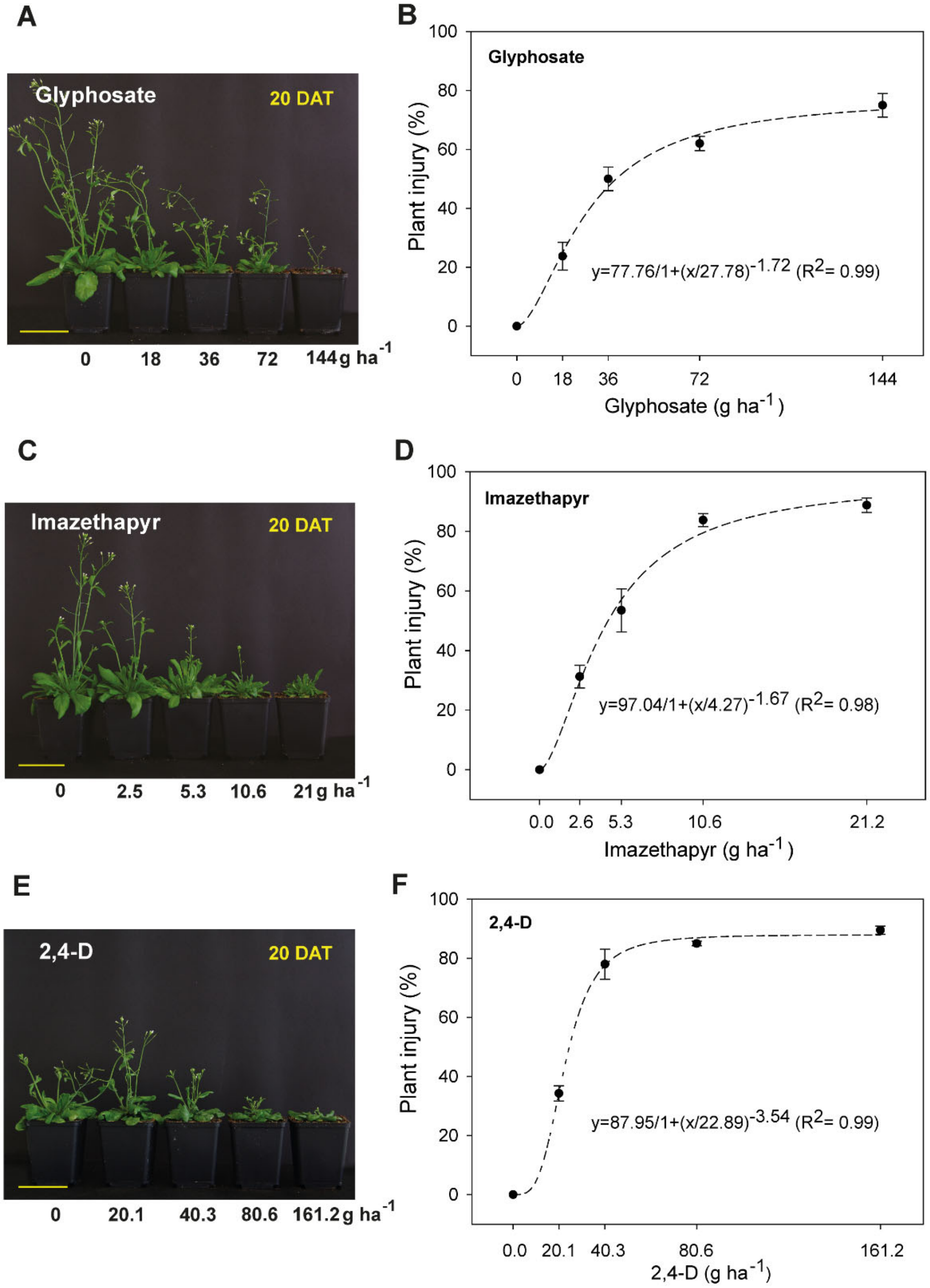

Figure S2. Shoot dry weight (g plant^{-1}) of *A. thaliana* Col-0 wild type (WT) 20 days after treatment (DAT) of glyphosate (a), imazethapyr (b) and 2,4-D (c). The graphs were plotted with the average and the vertical bars indicate the confidence interval. Regression analysis was performed, fitted to a non-linear logistic model with three parameters [$y= a/1+(x/x0)^b$], proposed by Streibig (1988) [60].

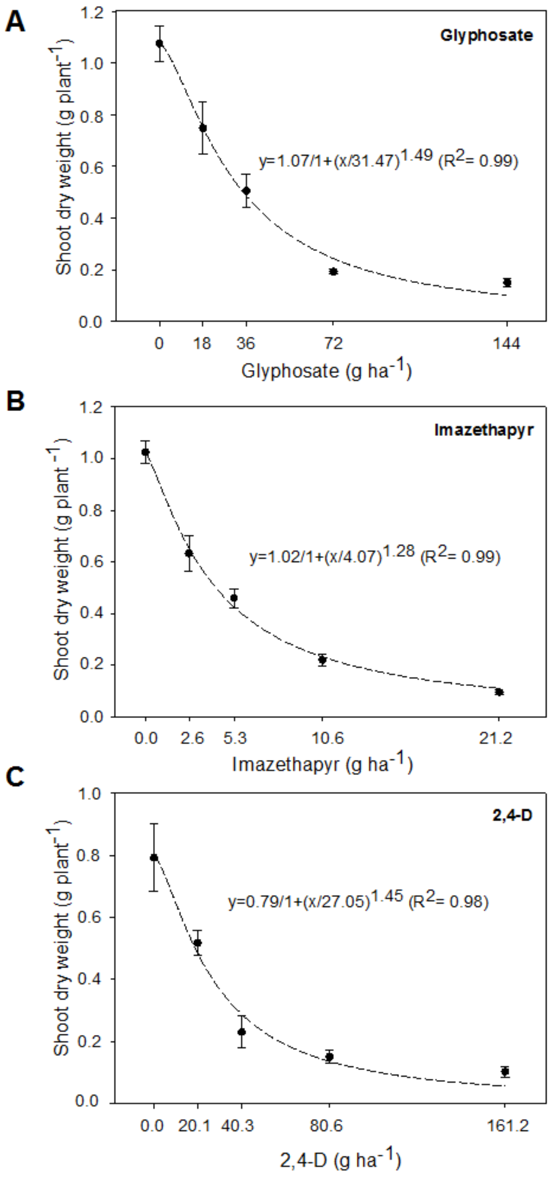

Figure S3. Leaf length (% untreated check) of *A. thaliana* Col-0 wild type (WT) and mutants, at 10 days after treatment (DAT) of glyphosate (a), imazethapyr (b) and 2,4-D (c). Means of studies one and two followed by different letters differ significantly according to Tukey's test ($p \leq 0.05$); ns=non-significant. Mutant followed by asterisk differ significantly from WT according to Tukey's test ($p \leq 0.05$).

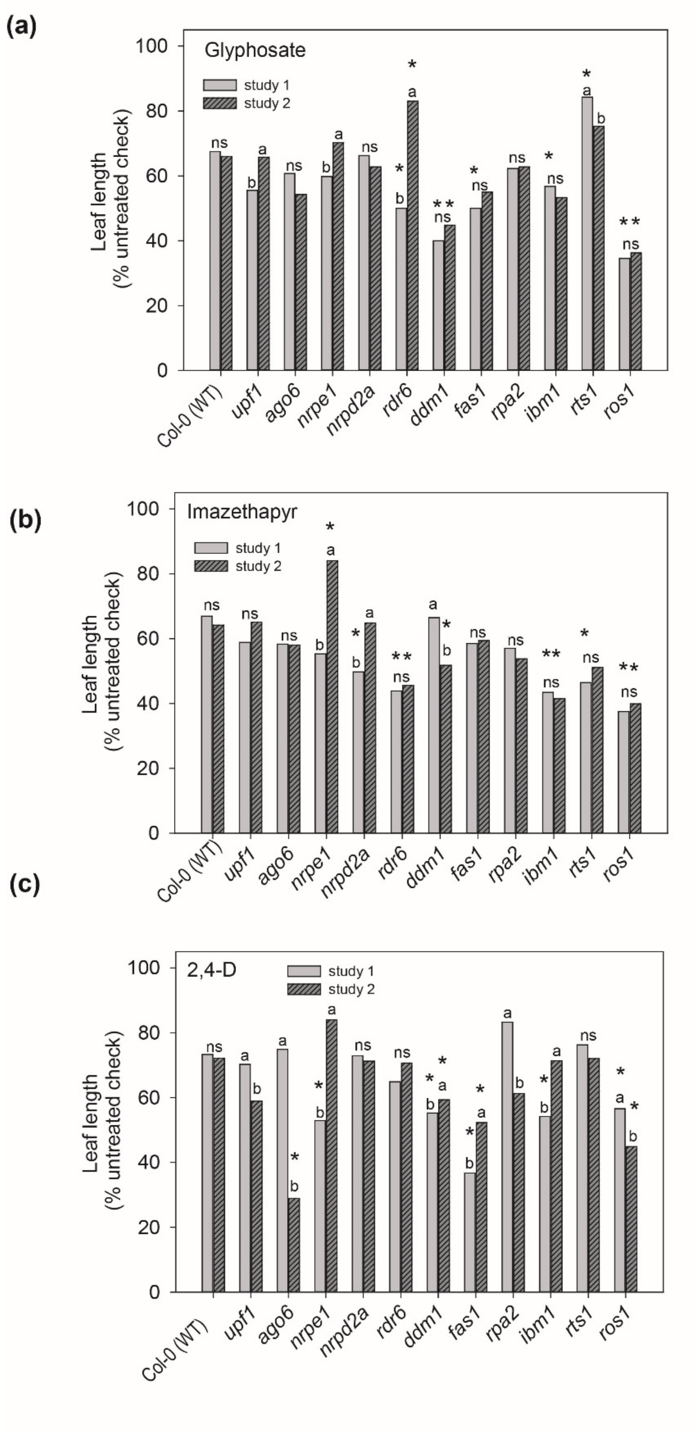

Figure S4. Bioinformatic analysis of RNA-seq data by using CummeRbund plots. (a) Expression level distribution for all genes in wild type (WT) and *ros1* mutant, in control (mock) condition and imazethapyr treatment (IM); FPKM, fragments per kilobase of transcript per million fragments mapped reads. (b) Volcano plots showing significant (adjusted p-value <0.05) differentially expressed genes, in red color.

(a)

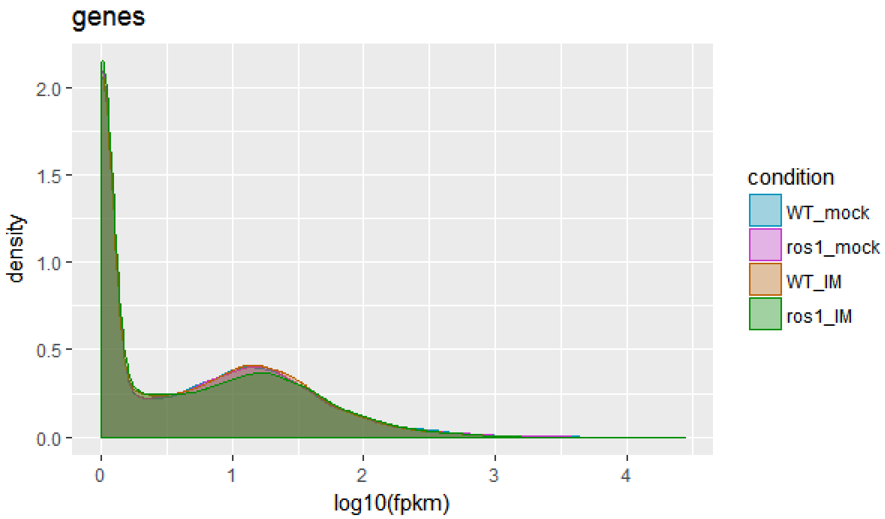

(b)

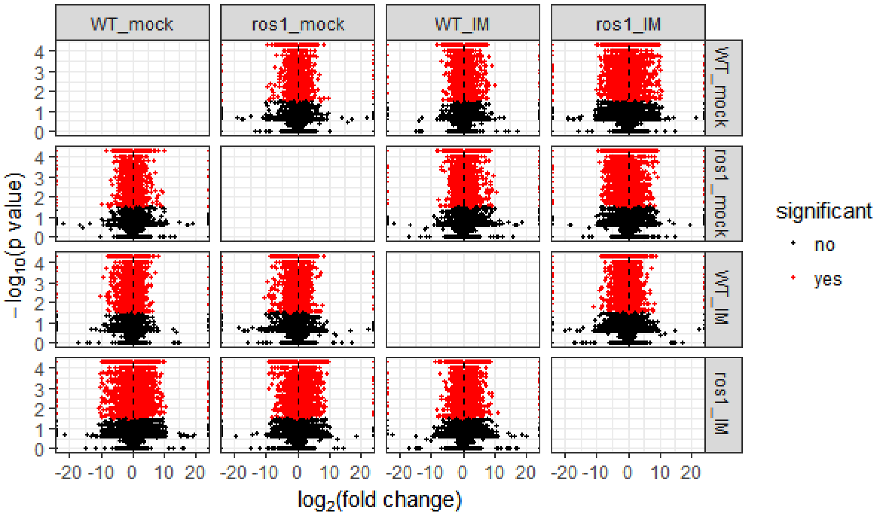

Figure S5. Pie chart representing Gene Ontology (GO) of up-regulated genes in Arabidopsis wild type (WT) 48 hours after imazethapyr (IM) treatment. 2375 differential expression of genes (DEGs) were annotated in at least one of the three GO categories: cellular component, biological process and molecular function.

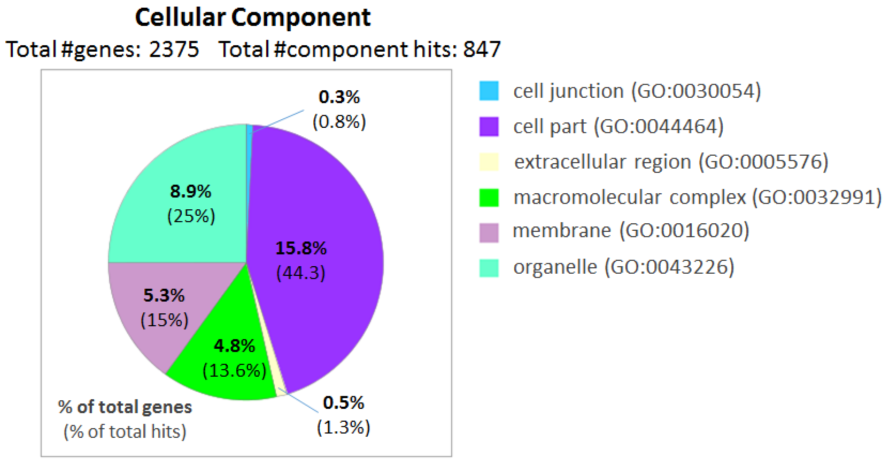

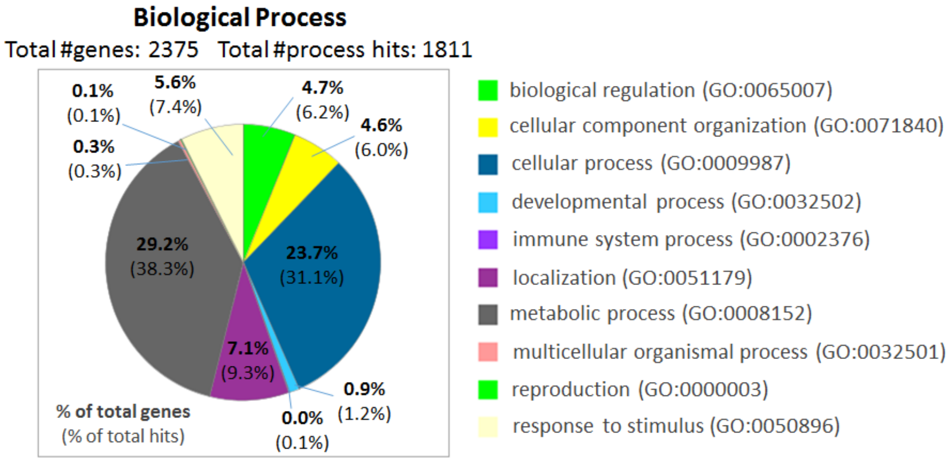

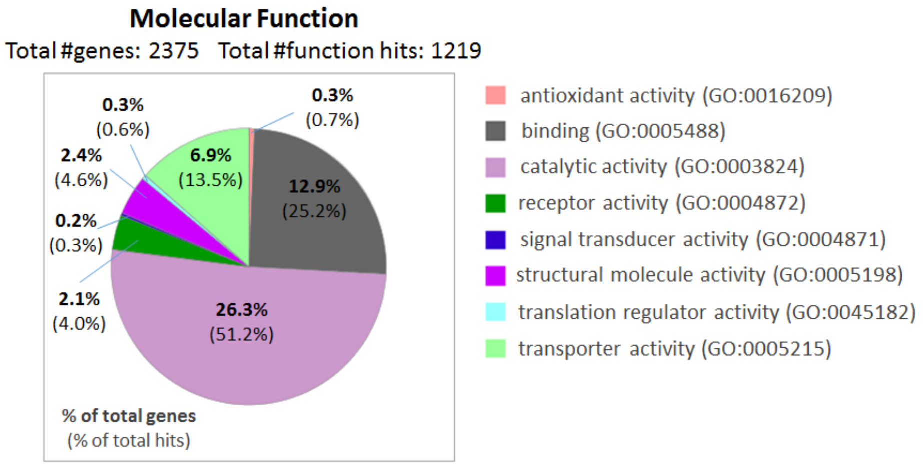

Figure S6. Detailed information of biological process representing the percent of genes involved in biological process pathways of up-regulated in wild type (WT) 48 hours after imazethapyr (IM) treatment, performed by using singular enrichment analysis (SEA) in AgriGo. Gray and black bars indicate the percent of genes related to the input list and the percent of genes compared to genome reference, respectively.

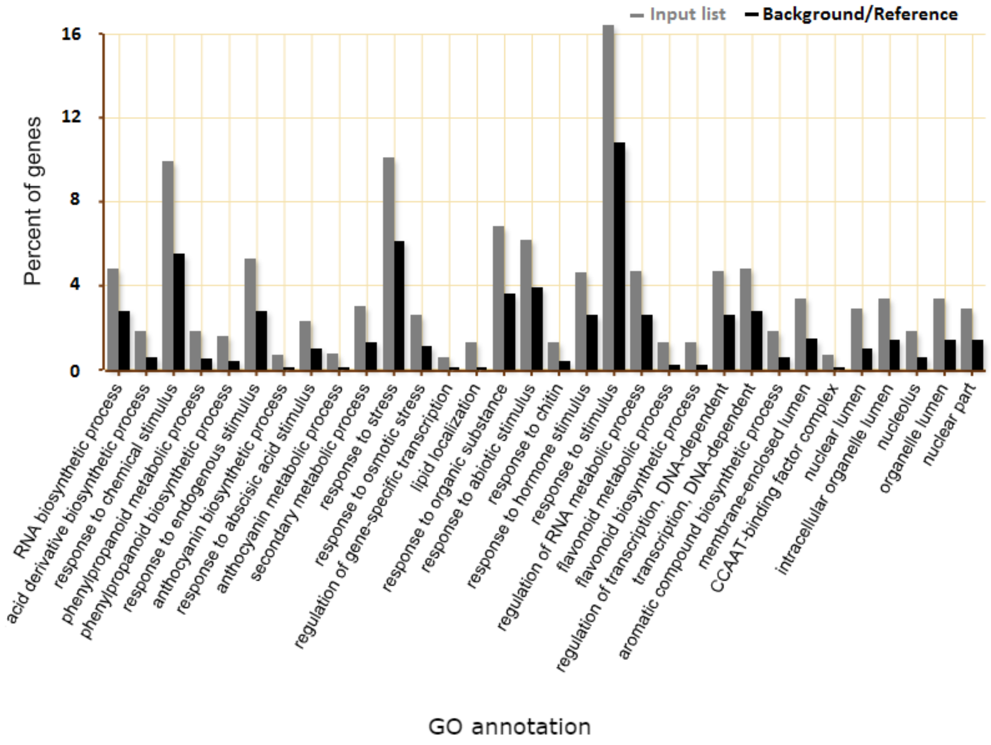

Figure S7. Pie chart representing Gene Ontology (GO) of down-regulated genes in *A. thaliana* Col-0 wild type (WT) 48 hours after imazethapyr (IM) treatment. 1164 differential expression of genes (DEGs) were annotated in at least one of the three GO categories: cellular component, biological process, and molecular function.

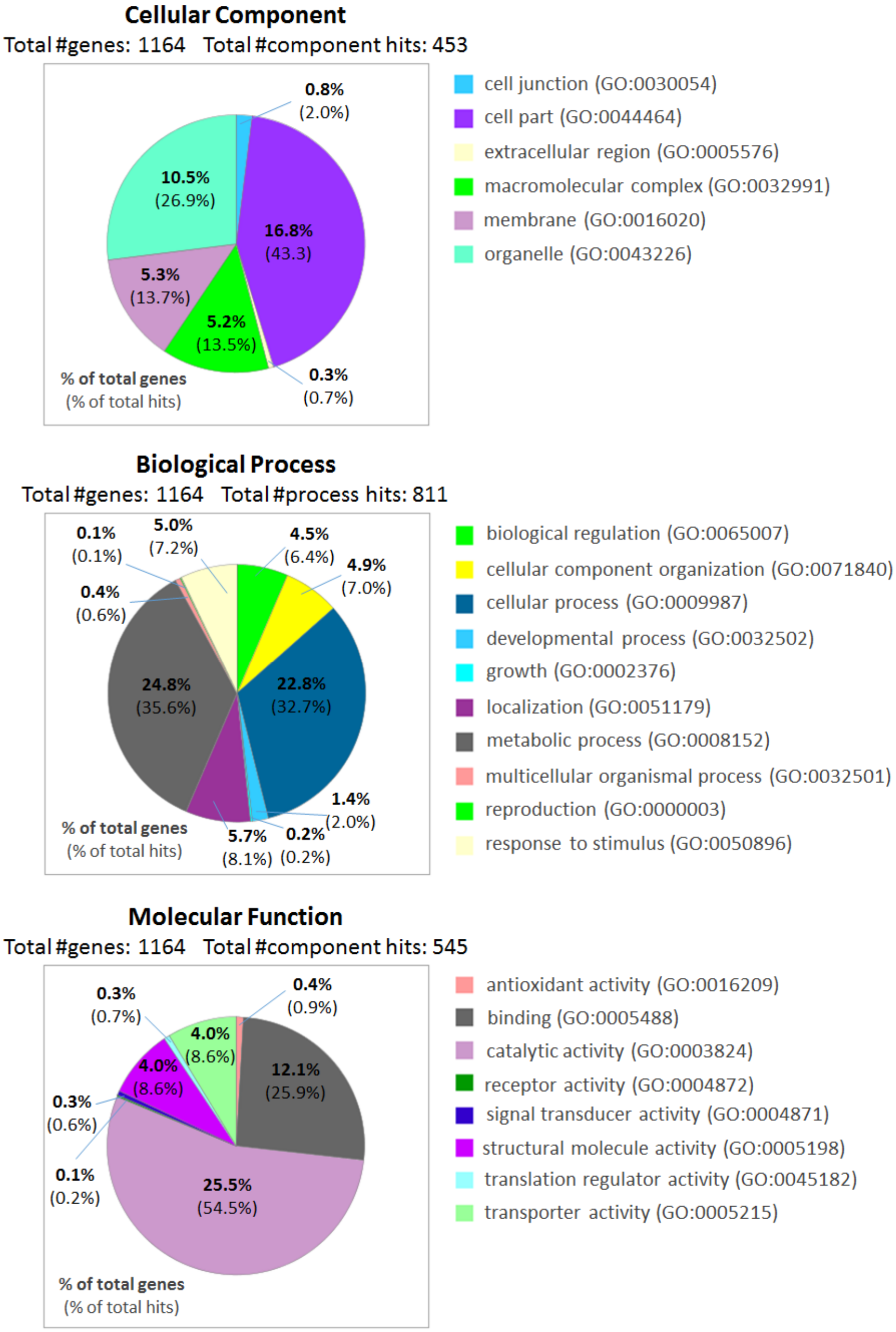

Figure S8. Detailed information of biological process representing the percent of genes involved in biological process pathways of down-regulated in wild type (WT) 48 hours after imazethapyr (IM) treatment, performed by using singular enrichment analysis (SEA) in AgriGo. Gray and black bars indicate the percent of genes related to the input list and the percent of genes compared to genome reference, respectively.

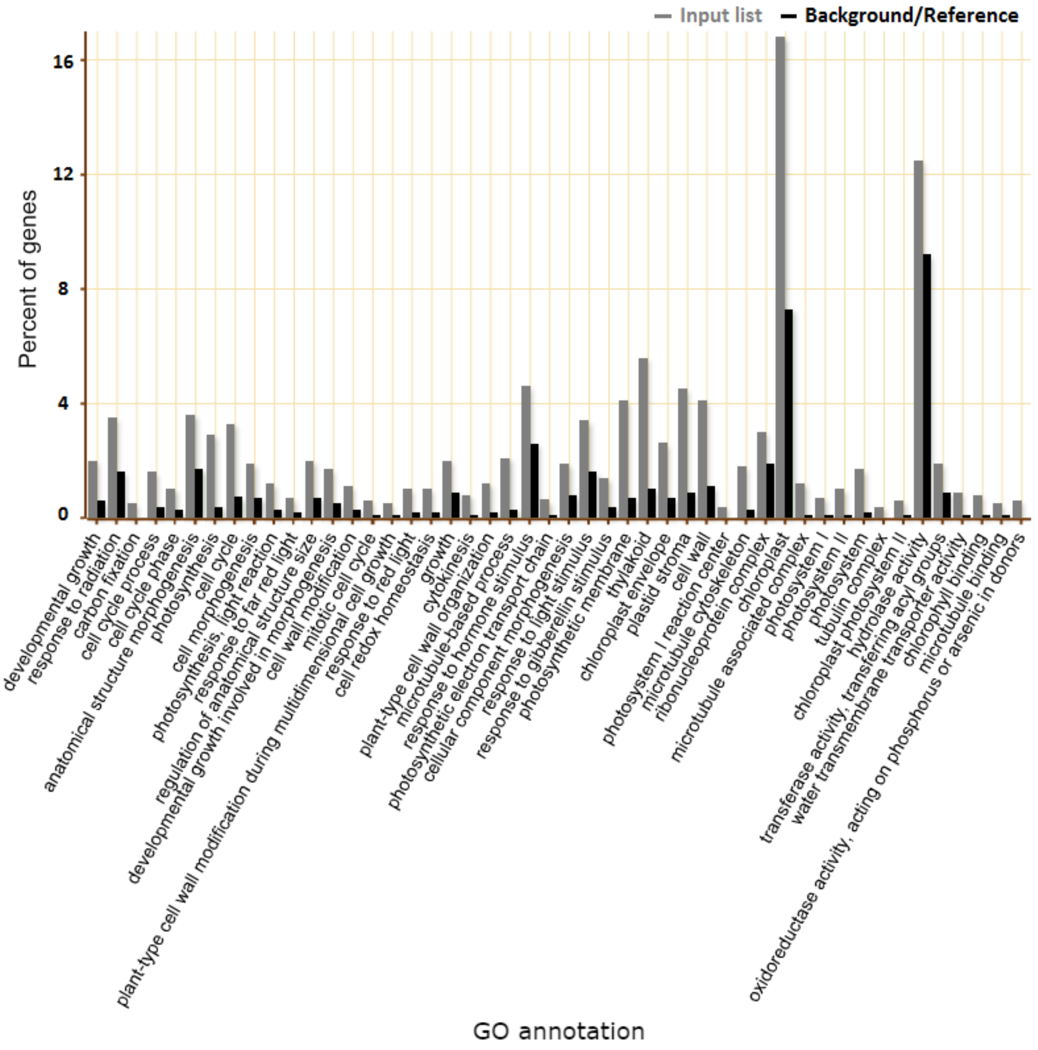

Figure S9. Pie chart representing Gene Ontology (GO) of 89 genes of interest induced by imazethapyr (IM) in *A. thaliana* wild type (WT) and down-regulated in *ros1* mutant. 75 differential expression of genes (DEGs) were annotated in at least one of the three GO categories: cellular component biological process and molecular function.

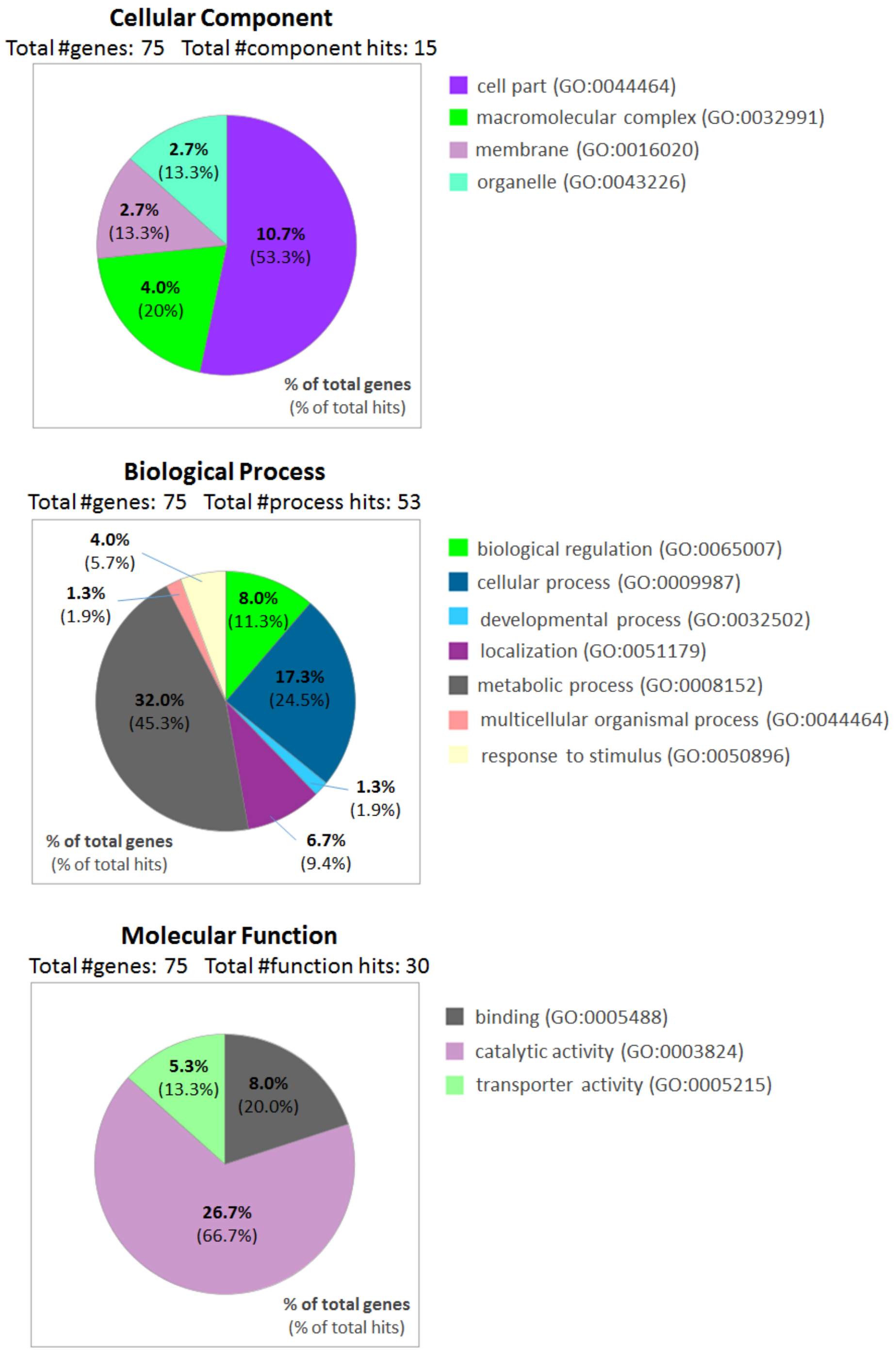

Figure S10. Part I of the overview of pathways overrepresented, according to singular enrichment analysis (SEA). The color scale indicates the significance levels of enrichment analysis. The arrows represent the relationship between parent-child terms.

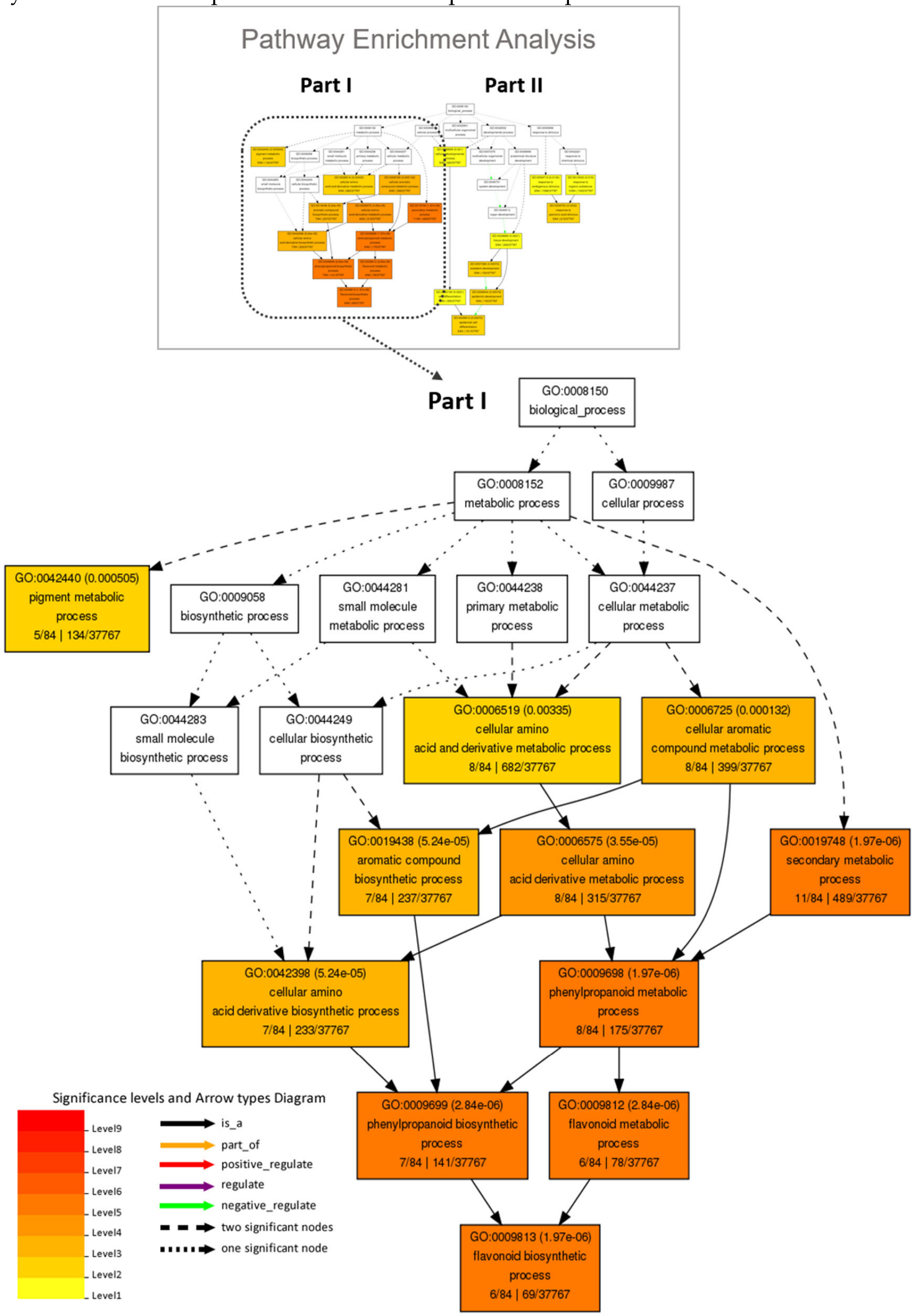

Figure S9. Part II of the overview of pathways overrepresented, according to singular enrichment analysis (SEA). The color scale indicates the significance levels of enrichment analysis. The arrows represent the relationship between parent-child terms.

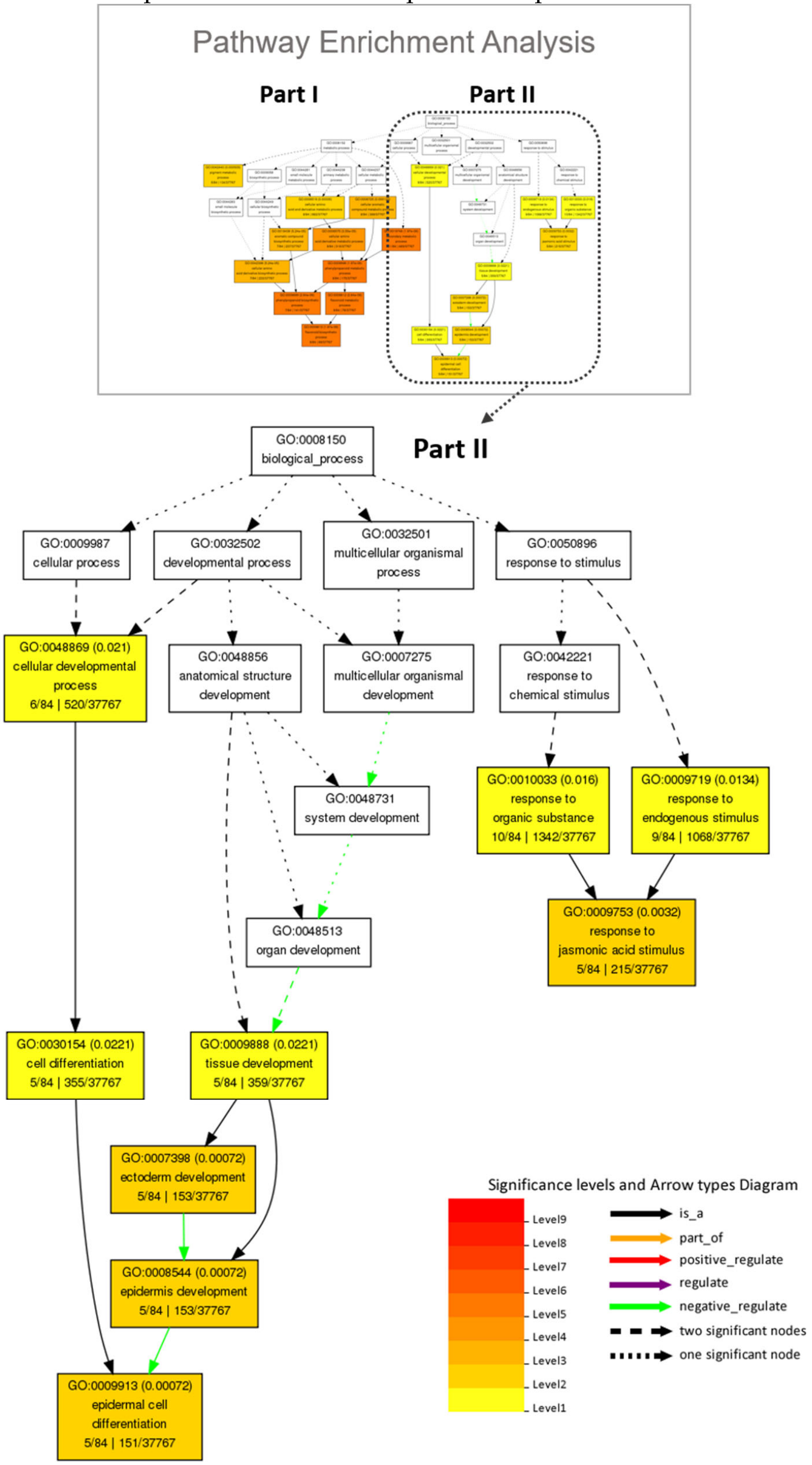

EXPERIMENTAL PROCEDURES

Table S5. Primers used in the analysis of transcription levels of GUS and for quantitative RT-PCR validation of genes by RNA-Seq.

Target	Primer Name	Sequence (5' → 3')	Product Size (bp)
GUS	GUS_F	TTAACTATGCCGGAATCCATCGC	128
	GUS_R	CACCACCTGCCAGTCAACAGACGC	
GAPC-2	GAPC-2_F	ATCGGTCGTTTGGTTGCTAGAGT	251
	GAPC-2_R	ACAAAGTCAGCTCCAGCCTCA	
UBC28	UBC28qF	TCCAGAAGGATCCTCCAACTTCCTGCAG T	124
	UBC28qR	ATGGTTACGAGAAAGACACCGCCTGAAT A	
TT7	TT7q_F	CTCGCCGGAGTATTCAACAT	143
	TT7q_R	CCGTTCATTTCGTGCTCTTT	
HMTDSP	HMTDSPq_F	AGCCTCAACCACCTCAGAAG	110
	HMTDSPq_R	AGCGGAAGACGAAACAAGAA	
SCAMP	SCAMPq_F	CCACCAGTCATCTTCCAAGG	113
	SCAMPq_R	CAGAAGAACCCAGCACCAAT	
MFSP	MFSPq_F	CCCCAACCAAGTTCTTGAAA	124
	MFSPq_R	AATGTGGTCGCTCTTCTTGG	
ESTERASE	esterase_F	CAAATTTGAATCCTCTTCGTGA	126
	esterase_F	GCGAATACTTCTCCGACGAAT	
XTH10	XTH10q_F	AGAAACCATGGGGAGAAAGG	130
	XTH10q_R	ATGGACCCTTTGACCAATCA	
